# Bisphenol A and the risk of cardiometabolic disorders: a systematic review with meta-analysis of the epidemiological evidence

**DOI:** 10.1186/s12940-015-0036-5

**Published:** 2015-05-31

**Authors:** Fanny Rancière, Jasmine G. Lyons, Venurs H.Y. Loh, Jérémie Botton, Tamara Galloway, Tiange Wang, Jonathan E. Shaw, Dianna J. Magliano

**Affiliations:** Inserm, U1153, Epidemiology and Biostatistics Sorbonne Paris Cité Research Centre (CRESS), Early Origin of the Child’s Health and Development (ORCHAD) Team, Villejuif, France; Univ Paris Descartes, UMR1153, Paris, France; Department of Clinical Diabetes and Epidemiology, Baker IDI Heart and Diabetes Institute, Level 4, 99 Commercial Road, Melbourne, VIC 3000 Australia; Faculty of Pharmacy, Univ Paris-Sud, Châtenay-Malabry, France; Department of Biosciences, University of Exeter, College of Life and Environmental Sciences, Exeter, UK; Shanghai Clinical Center for Endocrine and Metabolic Diseases, Rui Jin Hospital, Shanghai Jiao Tong University School of Medicine, Shanghai, China

**Keywords:** Bisphenol A, Cardiovascular disease, Diabetes, Epidemiology, Hyperglycemia, Hypertension, Meta-analysis, Obesity, Overweight, Systematic review

## Abstract

Bisphenol A (BPA) is suspected to be associated with several chronic metabolic diseases. The aim of the present study was to review the epidemiological literature on the relation between BPA exposure and the risk of cardiometabolic disorders. PubMed and Embase databases were searched up to August 2014 by two independent investigators using standardized subject terms. We included observational studies (cohort, case–control and cross-sectional studies) carried out in children or adults, measuring urinary BPA (uBPA), including at least 100 participants and published in English. The health outcomes of interest were diabetes, hyperglycemia, measures of anthropometry, cardiovascular disease (CVD) and hypertension. Data were extracted and meta-analyzed when feasible, using a random-effects model. Thirty-three studies with sample size ranging from 239 to 4811 met the inclusion criteria, including five with a prospective design. Twelve studies reported on diabetes or hyperglycemia, 16 on anthropometry, 6 on CVD and 3 on hypertension. Evidence for a positive association between uBPA concentrations and diabetes, overweight, obesity, elevated waist circumference (WC), CVD and hypertension was found in 7/8, 2/7, 6/7, 5/5, 4/5 and 2/3 of the cross-sectional studies, respectively. We were able to conduct outcome-specific meta-analyses including 12 studies. When comparing the highest vs. the lowest uBPA concentrations, the pooled ORs were 1.47 (95 % CI: 1.21–1.80) for diabetes, 1.21 (95 % CI: 0.98–1.50) for overweight, 1.67 (95 % CI: 1.41–1.98) for obesity, 1.48 (95 % CI: 1.25–1.76) for elevated WC, and 1.41 (95 % CI: 1.12–1.79) for hypertension. Moreover, among the five prospective studies, 3 reported significant findings, relating BPA exposure to incident diabetes, incident coronary artery disease, and weight gain. To conclude, there is evidence from the large body of cross-sectional studies that individuals with higher uBPA concentrations are more likely to suffer from diabetes, general/abdominal obesity and hypertension than those with lower uBPA concentrations. Given the potential importance for public health, prospective cohort studies with proper adjustment for dietary characteristics and identification of critical windows of exposure are urgently needed to further improve knowledge about potential causal links between BPA exposure and the development of chronic disease.

**Electronic supplementary material:**

The online version of this article (doi:10.1186/s12940-015-0036-5) contains supplementary material, which is available to authorized users.

## Introduction

In recent decades, large and rapid increases in diabetes and obesity prevalence have been observed worldwide. Apart from traditional risk factors such as family history, sedentary lifestyle and energy dense dietary intake, attention has recently turned to environmental toxicants called endocrine-disrupting chemicals (EDCs) because of their ability to interfere with synthesis, secretion, transport, metabolism, binding action, or elimination of natural blood-borne hormones, and to induce obesogenic or diabetogenic effects [1]. In particular, bisphenol A (BPA) has been strongly suspected as a potential contributor to these disease aetiologies [2–5].

BPA is a synthetic monomer used in the manufacture of polycarbonate plastics and epoxy resins, with a world production estimated at 3.8 million tons in 2006 [6]. Importantly, this production of BPA is expected to increase further in the coming years, given the robust demand for polycarbonate plastics and epoxy resins from China [7] and other emerging markets. The primary source of human exposure to BPA is presumed to be *via* the ingestion of food which has been stored or reheated in BPA-lined containers, but recent data suggest there is at least some exposure from drinking water, dental sealants, thermal paper and, to a lesser extent, inhalation of household dust particles [8–12]. BPA is ubiquitous in our environment, as evidenced by the fact that over 90 % of individuals have detectable levels of BPA present in their urine [13], which is the primary route of excretion in humans [14]. BPA has been found in neonates, children and adults [13], and can be measured in a range of bodily fluids and tissues, including urine, blood, saliva, placental tissue, adipose tissue and breast milk [13, 15].

There is accumulating *in vitro* and animal data (small and large animal) supporting a role of BPA in the development of diabetes, supporting a role of BPA in the development of diabetes, cardiovascular disease (CVD) and obesity. BPA is structurally similar to 17β-estradiol and thus binds to estrogen-related receptors (ER) such as ERα, ERβ and ERγ, the G protein-coupled estrogen receptor GPR30, and the peroxisome proliferator-activated receptor gamma (PPAR-γ) [16, 17]. While the mechanisms of action are not fully understood, binding of BPA to these receptors has been shown to induce insulin resistance, adipogenesis, pancreatic beta-cell dysfunction, inflammation, and oxidative stress [3, 18–20]. At concentrations typically seen in humans, BPA has been shown to act *via* extranuclear ERα [21] and ERβ [22]. These two studies used β-cells and whole islets of Langerhans from mice lacking ERα and ERβ and humans to demonstrate that the low dose effect of BPA is mediated *via* these estrogen receptors. Other experimental studies have also shown that BPA at environmentally relevant doses could inhibit the release of adiponectin, an adipokine that protects humans from obesity-related metabolic syndrome [23]. It also may have direct pro-angiogenic effects on human primary endothelial cells, suggesting that the human endothelium may be an important target for BPA [24]. Recently, Marmugi et al. showed that BPA exposure for 8 months in adult mice resulted in increased adipose tissue mass, hyperglycaemia, glucose intolerance, hypercholesterolemia and increased cholesterol biosynthesis by the liver [25]. However, the relevance of animal studies to humans remains unclear due to enterohepatic recirculation in rodents, resulting in a slower rate of BPA excretion compared with humans [14].

Given the ongoing policy debate on the possible public health benefits of minimizing BPA exposure, there is an urgent need for research to adequately evaluate cardiometabolic health in relation to BPA exposure. The past 10 years have seen a rapid increase in published reports of human, population-based epidemiological studies linking BPA to obesity, diabetes and CVD, most of which are cross-sectional studies. To date, three reviews have evaluated the available literature concerning BPA and chronic disease in humans [26–28]. However, the authors did not perform meta-analysis and, as the body of literature is growing rapidly, important new evidence can appear within short timelines [29]. Thus the aims of the present review were:

1. to provide insight into the most recent epidemiological evidence on the association between urinary BPA (uBPA) concentrations and chronic, cardiometabolic disorders (diabetes, overweight, obesity, CVD and hypertension);
2. to carry out meta-analysis when feasible; and
3. to identify the gaps in research that will allow for a comprehensive assessment of possible risk to humans.

## Methods

This review follows PRISMA guidelines for systematic reviews of observational studies [30].

### Eligibility criteria

Studies were included if the following inclusion criteria were met:

- *Participants* – Human studies including adults or children (however only diabetes in adults, in an attempt to limit the analysis to type 2)
- *Outcomes* – Studies examining diabetes, hyperglycemia, measures of anthropometry/adiposity, CVD or hypertension. Surrogate cardiometabolic outcomes such as blood cholesterol levels and insulin sensitivity indices (e.g., Homeostasis Model Assessment of Insulin Resistance [HOMA], Quantitative Insulin Sensitivity Check Index [QUICKI], Matsuda Index) were not considered.
- *Exposure* – Urinary measures of BPA and/or BPA metabolites (studies examining BPA exposure in pregnant women in relation to outcome in the offspring were beyond the scope of this review)
- *Sample size* – Studies including at least 100 participants,
- *Language* – Studies published in English,
- *Appropriate statistical adjustment* – Models including adjustment for confounders (at least age and gender).

### Information sources and search

PubMed and Embase databases were searched to identify potential studies for this review published up to August 2014. The keywords (and corresponding Medical Subject Heading [MeSH] terms for the search in PubMed) ‘body mass index’, ‘overweight’, ‘obesity’, ‘waist circumference’, ‘body weight’, ‘abdominal obesity’, ‘cardiovascular disease’, ‘coronary heart disease’, ‘diabetes’, ‘hypertension’, ‘blood pressure’, ‘insulin resistance’, ‘glucose intolerance’ were combined with the Boolean operator ‘OR’. The key term ‘Bisphenol A’ was entered and combined with the former using the Boolean operator ‘AND’. The full electronic search strategy in PubMed is reported in the Additional file 1: Figure S1. References from identified studies were hand-searched to ensure that no relevant studies were missed.

### Study selection and data extraction

All the identified publications were evaluated for relevance by two independent reviewers (DJM, FR), on the basis of their titles and abstracts; any disagreement was resolved by discussion. Full texts of the selected abstracts were then checked for the inclusion criteria by the reviewers. Final eligibility of studies was decided by consensus.

Summary data for included studies were extracted into a standardized tool that included: study design, country, population, sample size, age, gender, ethnicity, outcome definition, method of exposure measurement, uBPA levels and categorization, results expressed as adjusted odds ratios (OR), hazard ratios (HR) or β-coefficients, and variables used in adjustment.

The quality of individual studies, with regard to the outcomes of interest, was independently assessed by two investigators. We utilised a scoring system based on the established OHAT guidelines [31] adapted to reflect the characteristics of the included studies: longitudinal design (2 points), population-based study (1 point), outcome assessment including measurements (1 point), collection of at least 2 urine samples per participant (1 point), control for urine dilution (1 point), adjustment for dietary intake (1 point), and adjustment for socioeconomic variables (1 point). Studies were then classified as ‘low quality’ (total score between 0 and 2), ‘medium quality’ (total score between 3 and 5), or ‘high quality’ (total score between 6 and 8).

### Meta-analysis

We appraised each study to examine sources of heterogeneity, including difference in clinical outcomes and exposure measurements. Participant overlap between related studies was examined. For studies that used similar sources of data (e.g., surveys of National Health and Nutrition Examination Survey [NHANES], over concurrent periods and similar age range), only the study with the largest data set was included in the meta-analysis.

For each outcome, two studies with comparable outcome and exposure definitions were sufficient to perform a meta-analysis. For anthropometric outcomes, we performed pooled and separate analysis for children and adults. ORs with corresponding confidence intervals (CIs) were extracted from the most adjusted model. For summary purposes, we pooled OR estimates comparing extreme categories of uBPA levels (the highest vs. lowest uBPA levels). A random effects model was used. The logarithm of the OR and its standard error (SE) was calculated using the formula of log (upper limit of 95 % CI) minus log (lower limit of 95 % CI) divided by 3.92 (2*1.96, the 97.5th percentile of the standard normal distribution) and was entered into REVMAN 5.1 software (REVMAN 2011). Heterogeneity between studies was tested using Chi-squared test and quantified by calculating the I^2^ statistic. We produced forest plots to visually assess the individual study ORs and overall ORs, with corresponding 95 % CIs.

## Results

### Study selection

**Fig. 1:**
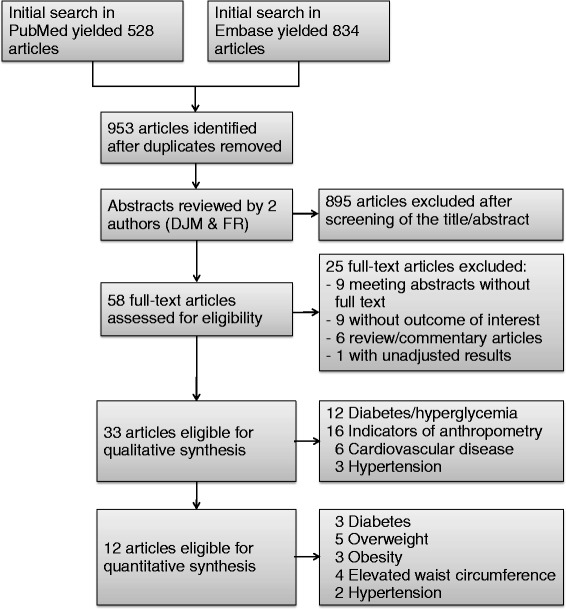
Flow diagram of the search strategy for the systematic review (some studies reported on several outcomes)

The initial search identified 953 studies in total. Of these, 895 studies were excluded after screening of the titles/abstracts (studies did not meet inclusion criteria e.g., were animal or *in vitro* studies, reported only serum BPA levels, or were review/commentary articles). Fifty eight studies were thus identified and reviewed in full-text versions, from which 33 studies were selected to be included in the systematic review (Fig. 1).

Twelve independent cross-sectional studies were selected for quantitative synthesis, making meta-analysis possible for 5 health outcomes: diabetes, overweight, obesity, elevated waist circumference (WC) and hypertension. Individual and pooled OR estimates are shown in the Additional file 1: Figure S2. Studies included and excluded from meta-analysis and reasons for exclusion are summarized in the Additional file 1: Table S1.

### Characteristics of studies

Characteristics for each of the 33 articles included in the review are summarized in Table 1 and detailed in the Additional file 1: Table S2.

**Table 1:** Summary characteristics of studies included in the systematic review (*n* = 33 studies)

Study characteristics	*n*
Year of publication	
2008	1
2009	0
2010	2
2011	5
2012	10
2013	8
2014	7
Geographical setting	
Asia	10
Europe	3
North America	20
Design	
Cross-sectional	28
Prospective	5
Age category	
Children	8
Adults	25
Gender	
Mixed	30
Women only	3
Sample size	
<500	5
500–1000	4
1001–2000	9
2001–3000	6
3001–4000	6
4001–5000	3
Health outcomes^a^	
Diabetes	9
Prediabetes	1
Hyperglycemia	2
Overweight	7
Obesity	7
Elevated waist circumference	5
Cardiovascular disease	6
Hypertension	3
Urinary sample	
Spot sample	27
First-morning-void sample	3
Second-morning void sample	1
12-h sample	1
24-h sample	1

^a^Some studies reported on several outcomes

#### Study design and population

This systematic review includes 28 cross-sectional and 5 longitudinal studies, drawn from 19 different study populations and 6 different countries. Sample sizes ranged from 239 [32] to 4811 [33] representing a total of 69,486 participants, not accounting for participant overlap across publications utilizing data from the same study populations.

Among the cross-sectional studies, 16 (57 %) used study populations from the NHANES study (cycles ranging from the years 2003 to 2010) [33–48]. Potential overlap between all publications using data from NHANES is summarized in the Additional file 1: Table S3. Five cross-sectional studies (18 %) used Chinese populations [49–53], including two studies using the same population sample from Songnan Community in Shanghai [51, 52]. Four cross-sectional studies (14 %) used a Korean population [54–57], while other single studies were conducted in Italians [58], Iranians [32], and British populations [59]. Of the 5 prospective studies, one was on the EPIC-Norfolk study (UK) [60] and 4 from U.S. cohorts, NHS and NHSII [61, 62], HOME [63] and CHAMACOS [64].

Of the 16 NHANES publications, 11 investigated adults [34, 36, 37, 39–46], while four restricted their study population to children/teenagers [35, 38, 47, 48]. One further study examined adults for CVD outcomes; however this study was excluded from the diabetes outcome analysis because the study population included a mixed sample of children and adults [33]. Of the 12 other cross-sectional studies, 10 were conducted in adult populations [32, 51–59], and 2 in school-age children [49, 50]. Three of the prospective studies were conducted in adults [60–62]. Of the two in children [63, 64], follow-up for the infants in the HOME study was at age 1 and 2 years [63], and for children from the CHAMACOS cohort at 5 and 9 years old [64].

All NHANES cycles as well as the NHS, NHSII and HOME cohorts comprised multiple ethnicities, but were predominantly non-Hispanic white. Mothers of children from the CHAMACOS cohort were mostly Latina. In 10 publications, all participants were Asian, from China [49–53], South Korea [54–57] or Iran [32].

#### Outcomes

This review included 12 publications on abnormal glucose tolerance (9, 2 and 1 with outcomes of diabetes, hyperglycemia and prediabetes, respectively), 16 publications on anthropometry (7 with an outcome of overweight, 7 with obesity and 5 with elevated WC), 6 publications on CVD and 3 on hypertension.

#### Urinary BPA assessment and levels

All the studies used total (unconjugated and conjugated) uBPA concentration as the exposure variable. The majority of studies relied on a single uBPA measurement, however 3 assessed BPA exposure using repeated measurements: 2 follow-up assessments in paediatric studies [63, 64], and up to 5 measurements during the study period in an adult study [54].

The highest uBPA mean concentration was reported in a study in children within the NHANES 2003/08 cycles [35], with a mean (SE) of 5.0 (0.3) ng/mL in boys and 4.6 (0.3) ng/mL in girls. The earliest NHANES (2003/2004) study had the highest mean levels of BPA, and median BPA concentration followed a downward trend across subsequent NHANES cycles [37]. The lowest uBPA concentration was reported in a cross-sectional study conducted in Chinese school children in 2011 [50], with a median (interquartile range, IQR) of 0.60 (0.20–1.37) ng/mL.

#### Covariates

Tables 2, 3 and 4 describe all variables used for the adjustment in the statistical analyses for each separate publication. All models were adjusted for at least age and gender. In order to correct for urine dilution, the majority of studies adjusted for an indicator of renal function, either urinary creatinine [33–40, 42, 45–48, 52, 55–57, 60–62, 64], estimated glomerular filtration rate (eGFR) [51], specific gravity [50], or serum creatinine [32]. Four studies corrected for renal function using the ratio of BPA-to-creatinine as the exposure variable [34, 54, 63, 64]. All but 4 adult studies adjusted for smoking [32, 51, 53, 58], and in the studies of children, smoking exposure was assessed by urine cotinine [35, 38, 47, 48, 63, 64] or questionnaire [64]. For the studies that investigated glucose, CVD or hypertension as the outcome, all except one [34] adjusted for WC [33, 37, 40, 46, 51], body mass index (BMI) [32, 33, 37–41, 43, 45, 46, 56, 59–61] or both height and weight [54].

**Table 2:** Summary of results in studies used as primary data: diabetes, prediabetes and hyperglycemia (*n* = 12 publications)

Reference	Outcomes & definitions used	Urinary BPA categorisation	Main results	Adjustment in model(s) used for review
Prevalent diabetes (8 publications)				
Ahmadkhaniha et al. 2014 [32]	Type 2 diabetes: self-reported and doctor-diagnosed T2D according to the ADA guideline (FPG >126 mg/dL, HbA1c >6.5 %) for more than one year	BPA in two groups based on the median (<0.85 and ≥0.85 μg/L)	OR = 57.6 (21.1–157.05)	Age, sex, BMI, hypertension, serum triglyceride level, serum cholesterol level, serum creatinine (smoking and consumption of sugared drinks in plastic bottles or canned food in two past weeks were exclusion criteria)
Casey & Neidell 2013 [37]	Diabetes: self-report of doctor diagnosis	BPA continuous (not log-transformed)	Per SD increase:	Age, sex, urinary creatinine concentration, race/ethnicity, income, smoking, body mass index, waist circumference, veteran/military status, citizenship status, marital status, household size, pregnancy status, language at subject interview, health insurance coverage, employment status in the prior week, consumption of bottled water in the past 24 h, consumption of alcohol, annual consumption of tuna fish, presence of emotional support in one’s life, being on a diet, using a water treatment device, access to a routine source of health care, vaccinated for Hepatitis A or B, consumption of dietary supplements (vitamins or minerals), inability to purchase balanced meals on a consistent basis + survey cycle for pooled analyses
			2003/04: OR = 1.398 (1.183–1.653)	
			2005/06: OR = 1.008 (0.861–1.181)	
			2007/08: OR = 0.716 (0.500–1.025)	
			Pooled 2003/08: OR = 1.065 (0.973–1.166)	
		BPA continuous (log-transformed)	Per 10-fold increase:	
			2003/04: OR = 1.492 (1.267–1.757)	
			2005/06: OR = 1.230 (0.894–1.694)	
			2007/08: OR = 0.932 (0.759–1.146)	
			Pooled 2003/08: OR = 1.202 (1.049–1.377)	
		BPA in quartiles (ng/mL): Q1: <1.2; Q2: 1.2–2.2; Q3: 2.3–4.2; Q4: >4.2	Pooled 2003/08:	
			Q2 vs. Q1: OR = 1.443 (0.982–2.119)	
			Q3 vs. Q1: OR = 1.512 (0.998–2.289)	
			Q4 vs. Q1: OR = 1.760 (1.137–2.724)	
Kim & Park 2013 [56]	Type 2 diabetes: self-reported and doctor-diagnosed	BPA in quartiles (ng/mL): Q1: <1.36; Q2: 1.36–2.14; Q3: 2.15–3.32; Q4: >3.32	Q2 vs. Q1: OR = 1.23 (0.62–2.43)	Urinary creatinine, age, sex, BMI, education, cigarette smoking, income, place of residence
			Q3 vs. Q1: OR = 1.17 (0.60–2.28)	
			Q4 vs. Q1: OR = 1.71 (0.89–3.26)	
			p for trend = 0.374	
Lang et al. 2008 [39]	Diabetes: self-report of doctor diagnosis	BPA continuous	Per SD increase:	Age, sex, race/ethnicity, education, income, BMI, WC, smoking status, urinary creatinine
			OR = 1.39 (1.21–1.60)	
Melzer et al. 2010 [40]	Diabetes: self-report of doctor diagnosis	BPA continuous	Per SD increase:	Age, gender, ethnicity, education, income, BMI, WC, smoking status, urinary creatinine
			2003/04: OR = 1.40 (1.25–1.56)	
			2005/06: OR = 1.02 (0.76–1.38)	
			Pooled 2003/06: OR = 1.24 (1.10–1.40)	
Ning et al. 2011 [51]	Type 2 diabetes: FPG ≥7.0 mmol/L or plasma glucose ≥11.1 mmol/L two hours after oral glucose tolerance test or use of diabetes medication	BPA in quartiles (ng/mL): Q1: ≤0.47; Q2: 0.48 –0.81; Q3: 0.82–1.43; Q4: >1.43	Q2 vs. Q1: OR = 1.30 (1.03–1.64)	Age, sex, educational level, family history of diabetes, WC, systolic blood pressure, ln(TG level), ln(hsCRP level), ln(ALT level), estimated glomerular filtration rate, albumin level, total bilirubin level
			Q3 vs. Q1: OR = 1.09 (0.86–1.39)	
			Q4 vs. Q1: OR = 1.37 (1.08–1.74)	
			p for trend not statistically significant	
Shankar & Teppala 2011 [42]	Diabetes: fasting serum glucose >126 mg/dL or non-fasting serum glucose >200 mg/dL or HbA1c >6.5 % or self-reported current use of oral hypoglycemic medication or insulin	BPA in quartiles (ng/mL): Q1: <1.10; Q2: 1.10–2.10; Q3: 2.11–4.20; Q4: >4.20	Q2 vs. Q1: OR = 1.42 (1.03–1.96)	Age, gender, race-ethnicity, education categories, smoking, alcohol intake, BMI, systolic and diastolic blood pressure, urinary creatinine, total cholesterol
			Q3 vs. Q1: OR = 1.48 (1.05–2.08)	
			Q4 vs. Q1: OR = 1.68 (1.22–2.30)	
			p for trend = 0.002	
			Normal weight participants:	
			Q4 vs. Q1: OR = 3.17 (1.23–8.18)	
			Overweight/obese participants:	
			Q4 vs. Q1: OR = 1.56 (1.09–2.24)	
Silver et al. 2011 [46]	Type 2 diabetes: HbA1c ≥6.5 % or self-reported use of diabetes medication (insulin or blood sugar-lowering pills)	BPA continuous (log-transformed)	For a doubling in uBPA concentration:	Age, age^2^, urinary creatinine as natural splines with 4° of freedom, gender, race-ethnicity, education, household income, BMI, WC, smoking status
			2003/04: OR = 1.23 (1.07–1.41)	
			2005/06: OR = 1.06 (0.95–1.19)	
			2007/08: OR = 1.06 (0.91–1.23)	
			Pooled 2003/08: OR = 1.08 (1.02–1.16)	
Incident diabetes (1 publication)				
Sun et al. 2014 [61]	Type 2 diabetes: self-reported diagnosis confirmed with one of the ADA 1998 criteria: a) elevated glucose concentration and ≥1 symptom related to diabetes; b) no symptoms but elevated glucose concentrations on 2 separate occasions; or c) treatment with insulin or oral hypoglycemic medication	BPA in quartiles (ng/mL):	NHS cohort (older women):	Age at urine sample collection, ethnicity, fasting status, time of sample collection, menopausal status, use of hormone replacement therapy (NHSII only), urinary creatinine levels, smoking status, postmenopausal hormone use (NHS only), oral contraceptive use (NHSII only), physical activity, alcohol use, family history of diabetes, history of hypercholesterolemia or hypertension, Alternative Health Eating Index score, BMI
		NHS cohort / NHSII cohort	Q2 vs. Q1: OR = 0.91 (0.56–1.48)	
		Q1: <1.0 / <1.3	Q3 vs. Q1: OR = 0.98 (0.60–1.61)	
		Q2: 1.0–1.5 / 1.3–2.0	Q4 vs. Q1: OR = 0.81 (0.48–1.38)	
		Q3: 1.5–2.7 / 2.0–3.5	p for trend = 0.45	
		Q4: >2.7 / >3.5		
			NHSII cohort (younger women):	
			Q2 vs. Q1: OR = 1.34 (0.70–2.27)	
			Q3 vs. Q1: OR = 1.91 (1.11–3.29)	
			Q4 vs. Q1: OR = 2.08 (1.17–3.69)	
			p for trend = 0.02	
Prevalent prediabetes (1 publication)				
Sabanayagam et al. 2013 [41]	Prediabetes: FPG = 100-125 mg/dL or 2-h glucose concentration = 140–199 mg/dL or HbA1c = 5.7–6.4 % (ADA guidelines)	BPA in tertiles (ng/mL): T1: <1.3; T2: 1.3–3.2; T3: >3.2	T2 vs. T1: OR = 1.42 (1.14–1.76)	Age, gender, race-ethnicity, education, smoking, alcohol intake, BMI, physical inactivity, mean arterial blood pressure, C-reactive protein, total cholesterol/HDL ratio
			T3 vs. T1: OR = 1.34 (1.03–1.73)	
			p for trend = 0.02	
			Stronger associations among women and obese participants.	
			Women:	
			T2 vs. T1: OR = 1.36 (0.96–1.91)	
			T3 vs. T1: OR = 1.49 (1.00–2.22)	
			p for trend = 0.04	
			Obese:	
			T2 vs. T1: OR = 1.71 (1.05–2.80)	
			T3 vs. T1: OR = 1.65 (1.04–2.80)	
			p for trend = 0.04	
Prevalent hyperglycemia (2 publications)				
Beydoun et al. 2014 [34]	Hyperglycemia: FPG ≥100 mg/dL	BPA continuous (log-transformed)	OR = 1.0 (0.9–1.2)	Age, sex, race, education, marital status, smoking status, physical activity, dietary energy intake, urinary creatinine, survey wave
		BPA in quartiles (ng/mL): Q1: 0.3 to <1.0; Q2: 1.0 to <2.0; Q3: 2.0 to <3.7; Q4: ≥3.7	Q2 vs. Q1: OR = 0.9 (0.52–1.42)	
			Q3 vs. Q1: OR = 1.2 (0.7–1.9)	
			Q4 vs. Q1: OR = 1.1 (0.6–1.9)	
			p for trend = 0.55	
		Ratio of BPA-to-creatinine continuous (log-transformed)	OR = 1.0 (0.9–1.2)	
		Ratio of BPA-to-creatinine in quartiles: Q1: 0.001 to <0.01; Q2: 0.01 to <0.02; Q3: 0.02 to <0.03; Q4: ≥0.03	Q2 vs. Q1: OR = 1.1 (0.8–1.6)	
			Q3 vs. Q1: OR = 1.3 (0.8–1.9)	
			Q4 vs. Q1: OR = 1.2 (0.8–1.9)	
			p for trend = 0.30	
Eng et al. 2013 [38]	Abnormal glucose: FPG ≥100 mg/dL	BPA in quartiles (ng/mL)		Age, gender, race/ethnicity, urine creatinine, poverty-to-income ratio, serum cotinine as a marker of smoking status, soda consumption, BMI percentile
		Q1: <1.3	Q2 vs. Q1: OR = 0.77 (0.33–1.78)	
		Q2: 1.3–2.6	Q3 vs. Q1: OR = 1.32 (0.57–3.04)	
		Q3: 2.6–4.9	Q4 vs. Q1: OR = 0.63 (0.22–1.82)	
		Q4: >4.9		

*ADA* American Diabetes Association; *ALT* alanine aminotransferase; *BMI* body mass index; *BPA* bisphenol A; *FPG* fasting plasma glucose; *HbA1c* glycated hemoglobin; *HDL* high density lipoprotein; *hsCRP* high sensitivity C-reactive protein; NHS Nurses' Health Study; *OR* odds ratio, *SD* standard deviation; *T2D* type 2 diabetes; *TG* triglycerides; *uBPA* urinary bisphenol A; *WC* waist circumference

**Table 3:** Summary of results in studies used as primary data: indicators of anthropometry and adiposity (*n* = 16 publications)

Reference	Outcomes & definitions used	Urinary BPA categorisation	Results	Adjustment in model(s) used for review
In children				
Prevalent overweight (4 publications)				
Eng et al. 2013 [38]	Overweight: BMI ≥85th percentile for age/gender	BPA in quartiles (ng/mL): Q1: <1.3; Q2: 1.3–2.6; Q3: 2.6–4.9; Q4: >4.9	Q2 vs. Q1: OR = 1.00 (0.74–1.36)	Age, gender, race/ethnicity, urine creatinine, poverty-to-income ratio, serum cotinine as a marker of smoking status, soda consumption
			Q3 vs. Q1: OR = 1.17 (0.89–1.54)	
			Q4 vs. Q1: OR = 1.07 (0.80–1.44)	
Harley et al. 2013 [64]	Overweight: BMI ≥85th percentile at 5 and 9 years of age	Ratio of BPA-to-creatinine level as continuous (log_2_- transformed) at 5 years	OR = 1.07 (0.90–1.28)	Maternal prepregnancy BMI, household income, maternal education level, maternal years of residence in the United States, child’s environmental tobacco smoke exposure, soda intake, fast food intake, and sweet consumption
		Ratio of BPA-to-creatinine level in 3 tertiles at 5 years (μg/g):	T2 vs. T1: OR = 0.80 (0.45–1.42)	
		T1: <LOD-2.4; T2: 2.4–4.5 μg/g; T3: 4.6–349.8 μg/g	T3 vs. T1: OR = 1.36 (0.75–2.47)	
		Ratio of BPA-to-creatinine level as continuous (log_2_- transformed) at 9 years	OR = 1.06 (0.85–1.33)	
		Ratio of BPA-to-creatinine level in 3 groups at 9 years (μg/g):	G2 vs. G1: OR = 3.08 (1.18–8.02)	
		G1: <LOD (<0.4); G2: detectable < median (0.4–1.8); G3: detectable > median (1.8–22.5)	G3 vs. G1: OR = 4.20 (1.60–11.02)	
Li et al. 2013 [49]	Overweight: age- and gender-specific weight >90th percentile of the underlying population	BPA in 2 classes (ng/mL): Low BPA level: <2 (reference); high BPA level: ≥2 (2 mg/L is about the median urine BPA level in the U.S. population)	Girls	Age, gender, school, residence, paternal and maternal education and overweight, playing video games, unbalanced diet, eating junk food, vegetables or fruit, depression scores, sports/activities
			All: OR=1.29 (0.83–2.01)	
			Age 9-12: OR= 2.32 (1.15-4.65)	
			Age>12: OR= 0.90 (0.48-1.72)	
			Boys	
			All: OR=0.82 (0.55–1.23)	
			Age 9-12: OR= 0.71 (0.34-1.45)	
			Age>12: OR= 0.87 (0.52-1.45)	
Trasande et al. 2012 [47]	Overweight: BMI z-score ≥1.036 (85th percentile for age/sex)	BPA continuous (log-transformed)	OR = 1.04 (0.92–1.18)	Sex, caloric intake, television watching, poverty to income ratio, parental education, serum cotinine level, urinary creatinine level, age, race/ethnicity categories
		BPA in quartiles (ng/mL): Q1: ≤1.5; Q2: 1.5–2.7; Q3: 2.8–5.5; Q4: >5.6	Q2 vs. Q1: OR = 1.26 (0.96–1.64)	
			Q3 vs. Q1: OR = 1.28 (0.98–1.66)	
			Q4 vs. Q1: OR = 1.26 (0.86–1.82)	
Prevalent obesity (3 publications)				
Bhandari et al. 2013 [35]	Obesity: BMI ≥ 95th percentile for age/gender	BPA continuous (log-transformed)	OR = 1.25 (1.09–1.43)	Age, sex, race/ethnicity, education, moderate activity, urinary creatinine, serum cotinine
		BPA in quartiles (ng/mL): Q1: <1.5; Q2: 1.5–2.7; Q3: 2.8–5.4; Q4: >5.4	Q2 vs. Q1: OR = 2.35 (1.56–3.53)	
			Q3 vs. Q1: OR = 1.78 (1.13–2.79)	
			Q4 vs. Q1: OR = 2.55 (1.65–3.95)	
			p for trend = 0.002	
			Stratified analyses by sex (p for interaction = 0.07): association of strong magnitude and statistically significant among boys	
Eng et al. 2013 [38]	Obesity: BMI ≥95th percentile for age/gender	BPA in quartiles (ng/mL): Q1: <1.3; Q2: 1.3–2.6; Q3: 2.6–4.9; Q4: >4.9	Q2 vs. Q1: OR = 1.73 (1.16–2.58)	Age, gender, race/ethnicity, urine creatinine, poverty-to-income ratio, serum cotinine as a marker of smoking status, soda consumption
			Q3 vs. Q1: OR = 1.63 (1.08–2.46)	
			Q4 vs. Q1: OR = 2.05 (1.38–3.04)	
Trasande et al. 2012 [47]	Obesity: BMI z-score ≥1.64 (95th percentile for age/sex)	BPA continuous (log-transformed)	Continuous: OR = 1.24 (1.08–1.44)	Sex, caloric intake, television watching, poverty-to-income ratio, parental education, serum cotinine level, urinary creatinine level, age, race/ethnicity categories
		BPA in quartiles (ng/mL): Q1: ≤1.5; Q2: 1.5–2.7; Q3: 2.8–5.5; Q4: >5.6	Q2 vs. Q1: OR = 2.24 (1.54–3.24)	
			Q3 vs. Q1: OR = 2.08 (1.46–2.96)	
			Q4 vs. Q1: OR = 2.57 (1.72–3.83)	
Prevalent elevated waist circumference				
Eng et al. 2013 [38]	Abnormal WC: WC ≥90th percentile for age/gender Abnormal WC-to-height ratio: WC-to-height ratio ≥0.5	BPA in quartiles (ng/mL): Q1: <1.3; Q2: 1.3–2.6; Q3: 2.6–4.9; Q4: >4.9	Abnormal WC	Age, gender, race/ethnicity, urine creatinine, poverty-to-income ratio, serum cotinine as a marker of smoking status, soda consumption
			Q2 vs. Q1: OR = 1.33 (0.90–1.97)	
			Q3 vs. Q1: OR = 1.16 (0.75–1.81)	
			Q4 vs. Q1: OR = 1.40 (0.91–2.15)	
			Abnormal WC-to-height ratio	
			Q2 vs. Q1: OR = 1.37 (0.97–1.92)	
			Q3 vs. Q1: OR = 1.41 (1.07–1.87)	
			Q4 vs. Q1: OR = 1.56 (1.11–2.17)	
Other				
Braun et al. 2014 [63]	Change in BMI z-score between 2 and 5 years of age, as continuous	Ratio of BPA-to-creatinine continuous (log_10_-transformed)	Per 10-fold increase: β = −0.2 (−0.6, 0.1)	Maternal race, marital status, parity, age at delivery, household income, education, employment, insurance, BMI at 16 weeks of pregnancy, depressive symptoms at baseline, prenatal serum cotinine
		Ratio of BPA-to-creatinine in tertiles (μg/g creatinine):	T2 vs. T1: β = 0.0 (−0.3, 0.3)	
		T1: 2.1–11; T2: 11–20; T3: 20–314	T3 vs. T1: β = −0.2 (−0.5, 0.1)	
Eng et al. 2013 [38]	Prevalent abnormal body fat: body fat ≥85th percentile for age/gender	BPA in quartiles (ng/mL): Q1: <1.3; Q2: 1.3–2.6; Q3: 2.6–4.9; Q4: >4.9	Q2 vs. Q1: OR = 4.85 (0.80–21.4)	Age, gender, race/ethnicity, urine creatinine, poverty-to-income ratio, serum cotinine as a marker of smoking status, soda consumption
			Q3 vs. Q1: OR = 5.36 (0.71–43.3)	
			Q4 vs. Q1: OR = 2.10 (0.24–17.8)	
Harley et al. 2013 [64]	Incident overweight: BMI ≥85th percentile at 9 years of age	Ratio of BPA-to-creatinine level at 5 years as continuous (log_2_- transformed)	OR = 1.02 (0.84–1.23)	Maternal prepregnancy BMI, household income, maternal education level, maternal years of residence in the United States, child’s environmental tobacco smoke exposure, soda intake, fast food intake, and sweet consumption at age 5 years
		Ratio of BPA-to-creatinine level at 5 years in tertiles (μg/g):	T2 vs. T1: 0.91 (0.48–1.73)	
		T1: <LOD-2.4; T2: 2.4–4.5; T3: 4.6–349.8	T3 vs. T1: 1.28 (0.65–2.51)	
Wang et al. 2012b [50]	Prevalent BMI as continuous (kg/m^2^)	BPA continuous (log-transformed and corrected for specific gravity)	β = 0.017 (0.002–0.032)	Age, sex
Wells et al. 2013 [48]	Prevalent waist-to-height ratio as continuous	BPA in quartiles (ng/mL): Q1: <1.2; Q2: 1.2–2.6; Q3: 2.6–5.1; Q4: >5.1	Change in waist-to-height ratio:	Urinary creatinine, age, sex, race/ethnicity, education, smoking status based on serum cotinine, caloric intake
			Q2 vs. Q1: β = 0.011 (0.001–0.020)	
			Q3 vs. Q1: β = 0.010 (0.001–0.019)	
			Q4 vs. Q1: β = 0.016 (0.007–0.026)	
			Significant associations among boys but not girls.	
In adults				
Prevalent overweight (3 publications)				
Carwile & Michels 2011 [36]	Overweight: 25 ≤ BMI < 30 kg/m^2^ (reference: BMI <25 kg/m^2^)	BPA in quartiles (ng/mL): Q1: ≤1.1; Q2: 1.2–2.3; Q3: 2.4–4.6; Q4: ≥4.7	Q2 vs. Q1: OR = 1.66 (1.21–2.27)	Age, gender, race/ethnicity, education, smoking status, urinary creatinine
			Q3 vs. Q1: OR = 1.26 (0.85–1.87)	
			Q4 vs. Q1: OR = 1.31 (0.80–2.14)	
Kim et al. 2011 [55]	Overweight: BMI = 23-24.9 kg/m^2^, according to the WHO definitions for the Asian populations (reference: BMI <18.5 kg/m^2^)	BPA continuous (log-transformed)	Adjusted proportional change (95 % CI) = 1.01 (0.78–1.31)	Age, gender, education, income, cigarette smoking status, place of residence, urinary creatinine
Wang et al. 2012a [52]	Generalized overweight: 24 ≤ BMI < 28 kg/m^2^, according to Chinese criteria (reference = BMI <24 kg/m^2^)	BPA in quartiles (ng/mL): Q1: ≤0.47; Q2: 0.48–0.81; Q3: 0.82–1.43; Q4: >1.43	Q2 vs. Q1: OR = 1.23 (0.97–1.57)	Age, sex, urinary creatinine, smoking, alcohol drinking, education levels, systolic blood pressure, HDL cholesterol, LDL cholesterol, total cholesterol, TG, hsCRP, fasting plasma glucose, fasting serum insulin, serum ALT and GGT
			Q3 vs. Q1: OR = 1.28 (1.01–1.63)	
			Q4 vs. Q1: OR = 1.24 (0.97–1.59)	
Prevalent obesity (4 publications)				
Carwile & Michels 2011 [36]	Obesity: BMI ≥30 kg/m^2^ (reference: BMI <25 kg/m^2^)	BPA in quartiles (ng/mL): Q1: ≤1.1; Q2: 1.2–2.3; Q3: 2.4–4.6; Q4: ≥4.7	Q2 vs. Q1: OR = 1.85 (1.22–2.79)	Age, gender, race/ethnicity, education, smoking status, urinary creatinine
			Q3 vs. Q1: OR = 1.60 (1.05–2.44)	
			Q4 vs. Q1: OR = 1.76 (1.06–2.94)	
Kim et al. 2011 [55]	Obesity: BMI ≥25 kg/m^2^, according to the WHO definitions for the Asian populations (reference = BMI <18.5 kg/m^2^)	BPA continuous (log-transformed)	Adjusted proportional change (95 % CI) = 0.96 (0.75–1.23)	Age, gender, education, income, cigarette smoking status, place of residence, urinary creatinine
Shankar et al. 2012 [44]	Obesity: BMI ≥30 kg/m^2^ (reference = BMI <30 kg/m^2^)	BPA in quartiles (ng/mL): Q1: <1.10; Q2: 1.10–2.10; Q3: 2.11–4.20; Q4: >4.20	Q2 vs. Q1: OR = 1.40 (1.10–1.76)	Age, gender, race/ethnicity, education categories, smoking, alcohol consumption, physical inactivity, diabetes, hypertension, total cholesterol
			Q3 vs. Q1: OR = 1.59 (1.25–2.02)	
			Q4 vs. Q1: OR = 1.69 (1.30–2.20)	
			p for trend < 0.0001	
			Associations still significant in analyses stratified by sex.	
Wang et al. 2012a [52]	Generalized obesity: BMI ≥28 kg/m^2^, according to Chinese criteria (reference: BMI <28 kg/m^2^)	BPA in quartiles (ng/mL): Q1: ≤0.47; Q2: 0.48–0.81; Q3: 0.82–1.43; Q4: >1.43	Q2 vs. Q1: OR = 1.14 (0.87–1.50)	Age, sex, urinary creatinine, smoking, alcohol drinking, education levels, systolic blood pressure, HDL cholesterol, LDL cholesterol, total cholesterol, TG, hsCRP, fasting plasma glucose, fasting serum insulin, serum ALT and GGT
			Q3 vs. Q1: OR = 1.19 (0.90–1.57)	
			Q4 vs. Q1: OR = 1.50 (1.15–1.97)	
Prevalent elevated waist circumference (4 publications)				
Carwile & Michels 2011 [36]	Elevated WC: WC ≥102 cm in men and WC ≥88 cm in women	BPA in quartiles (ng/mL): Q1: ≤1.1; Q2: 1.2–2.3; Q3: 2.4–4.6; Q4: ≥4.7	Q2 vs. Q1: OR = 1.62 (1.11–2.36)	Age, gender, race/ethnicity, education, smoking status, urinary creatinine
			Q3 vs. Q1: OR = 1.39 (1.02–1.90)	
			Q4 vs. Q4: OR = 1.58 (1.03–2.42)	
Ko et al. 2014 [57]	Abdominal obesity: WC ≥90 cm in men and WC ≥85 cm in women	BPA in quartiles (μg/mL) Q1: <0.853; Q2: 0.853–1.407; Q3: 1.407–2.594; Q4: >2.594	Q2 vs. Q1: 1.117 (0.757–1.649)	Age, sex, urinary creatinine, education, income, alcohol consumption, smoking status
			Q3 vs. Q1: 1.337 (0.908–1.967)	
			Q4 vs. Q1: 1.938 (1.314–2.857)	
			p for trend = 0.01	
Shankar et al. 2012 [44]	Abdominal obesity: WC ≥102 cm in men and WC ≥88 cm in women	BPA in quartiles (ng/mL): Q1: <1.10; Q2: 1.10–2.10; Q3: 2.11–4.20; Q4: >4.20	Q2 vs. Q1: OR = 1.63 (1.20–2.22)	Age, gender, race/ethnicity, education categories, smoking, alcohol consumption, physical inactivity, diabetes, hypertension, total cholesterol
			Q3 vs. Q1: OR = 1.66 (1.28–2.14)	
			Q4 vs. Q1: OR = 1.59 (1.21–2.09)	
			p for trend = 0.0009	
			Associations still significant in analyses stratified by sex	
Wang et al. 2012a [52]	Abdominal obesity: WC ≥90 cm in men and WC ≥85 cm in women	BPA in quartiles (ng/mL): Q1: ≤0.47; Q2: 0.48–0.81; Q3: 0.82–1.43; Q4: >1.43	Q2 vs. Q1: OR = 1.26 (1.02–1.57)	Age, sex, urinary creatinine, smoking, alcohol drinking, education levels, systolic blood pressure, HDL cholesterol, LDL cholesterol, total cholesterol, TG, hsCRP, fasting plasma glucose, fasting serum insulin, serum ALT and GGT
			Q3 vs. Q1: OR = 1.28 (1.03–1.59)	
			Q4 vs. Q1: OR = 1.28 (1.03–1.60)	
Other				
Galloway et al. 2010 [58]	Prevalent WC as continuous Prevalent weight as continuous	Daily BPA excretion (μg/day) as a continuous variable	WC: β = 0.0062 (0.0016–0.0108)	Age, sex, study site
			Weight: β = 0.0064 (0.0023–0.0104)	
Kim et al. 2011 [55]	Prevalent normal weight: BMI = 18.5–22.9 kg/m^2^, according to the WHO definitions for the Asian populations (reference: BMI < 18.5 kg/m^2^)	BPA continuous (log-transformed)	Adjusted proportional change (95 % CI) = 0.92 (0.72–1.17)	Age, gender, education, income, cigarette smoking status, place of residence, urinary creatinine
Song et al. 2014 [62]	Weight change rate (WCR) during follow-up (kg/year)	BPA in quartiles (nmol/L):		Age at baseline, urinary creatinine concentration, cohort origin, menopausal status, smoking, alcohol consumption, physical activity, alternative healthy eating index, total energy intake
		Q1: median (IQR) = 3.6 (2.6–4.5)	Q2 vs. Q1: WCR = 0.15 (0.00–0.31)	
		Q2: median (IQR) = 6.4 (5.8–7.3)	Q3 vs. Q1: WCR = 0.18 (0.03–0.34)	
		Q3: median (IQR) = 10.5 (9.0–12.1)	Q4 vs. Q1: WCR = 0.23 (0.07–0.38)	
		Q4: median (IQR) = 21.9 (16.8–35.7)	p for trend = 0.02	
Zhao et al. 2012 [53]	Fat mass, fat-free mass, body weight, BMI, WC, hip circumference, waist-hip ratio (all variables as continuous)	BPA continuous	Fat mass: r = 0.35 (p < 0.001)	Age
			Fat-free mass: r = 0.186 (p = 0.009)	
			Body weight: r = 0.24 (p = 0.001)	
			BMI: r = 0.298 (p < 0.001)	
			WC: r = 0.296 (p < 0.001)	
			Hip circumference: r = 0.27 (p < 0.001)	
			Waist-hip ratio: r = 0.149 (p = 0.035)	
			With additional adjustment for age and BMI, BPA was still significantly associated with fat mass (r = 0.193, p = 0.006) but not with fat-free mass.	

*ALT* alanine aminotransferase; *BMI* body mass index; *BPA* bisphenol A; *GGT* gamma glutamyltransferase; *HDL* high density lipoprotein; *hsCRP* high sensitivity C-reactive protein; *IQR* interquartile range; *LDL* low density lipoprotein; *LOD* limit of detection; *OR* odds ratio; *TG* triglycerides; *WC* waist circumference; *WCR* weight change rate

**Table 4:** Summary of results in studies used as primary data: cardiovascular disease and hypertension (*n* = 9 publications)

Reference	Outcomes & definitions used	Urinary BPA categorisation	Results	Adjustment in model(s) used for review
Prevalent CVD (5 publications)				
Casey & Neidell 2013 [37]	CHD: self-report of doctor diagnosis	BPA continuous (not log-transformed)	Per SD increase:	Age, sex, urinary creatinine concentration, race/ethnicity, income, smoking, body mass index, waist circumference, veteran/military status, citizenship status, marital status, household size, pregnancy status, language at subject interview, health insurance coverage, employment status in the prior week, consumption of bottled water in the past 24 h, consumption of alcohol, annual consumption of tuna fish, presence of emotional support in one’s life, being on a diet, using a water treatment device, access to a routine source of health care, vaccinated for Hepatitis A or B, consumption of dietary supplements (vitamins or minerals), inability to purchase balanced meals on a consistent basis + survey cycle for pooled analyses
			2003/04: OR = 1.824 (1.288–2.583)	
			2005/06: OR = 1.267 (1.041–1.542)	
			2007/08: OR = 1.123 (0.854–1.476)	
			Pooled 2003/08: OR = 1.136 (1.014–1.273)	
		BPA continuous (log-transformed)	Per 10-fold increase:	
			2003/04: OR = 1.584 (1.066–2.354)	
			2005/06: OR = 1.178 (0.765–1.815)	
			2007/08: OR = 1.649 (1.025–2.654)	
			Pooled 2003/08: OR = 1.280 (0.993–1.649)	
		BPA in quartiles (ng/mL): Q1: <1.2; Q2: 1.2–2.2; Q3: 2.3–4.2; Q4: >4.2	Pooled 2003/08:	
			Q2 vs. Q1: 0.520 (0.250–1.084)	
			Q3 vs. Q1: 1.006 (0.508–1.994)	
			Q4 vs. Q1: 1.520 (0.774–2.987)	
Lakind et al. 2012 [33]	CHD: self-report of doctor diagnosis Heart attack: self-report of doctor diagnosis	BPA continuous	CHD, per unit increase:	Age, gender, ethnicity, education, income, smoking, heavy drinking, BMI, waist circumference, energy intake, family history of heart attack, hypertension, sedentary activity, total cholesterol, urinary creatinine. Pooled 2003/10 models were further adjusted for survey cycle, but were not adjusted for energy intake and sedentary activity.
			2003/04: OR = 1.03 (0.978–1.09)	
			2005/06: OR = 1.02 (0.996–1.04)	
			2007/08: OR = 0.996 (0.951–1.04)	
			2009/10: OR = 1.00 (0.998–1.01)	
			Pooled 2003/10: OR = 1.004 (0.998–1.009)	
			Heart attack, per unit increase:	
			2003/04: OR = 1.04 (0.996–1.09)	
			2005/06: OR = 1.02 (0.996–1.04)	
			2007/08: OR = 0.987 (0.941–1.04)	
			2009/10: OR = 1.00 (0.999–1.01)	
			Pooled 2003/10: OR = 1.002 (0.998–1.007)	
Lang et al. 2008 [39]	Heart attack, angina, CHD, CVD (any diagnoses of MI, angina or CHD), stroke; all self-reported doctor diagnoses	BPA continuous	Per SD increase:	Age, gender, race/ethnicity, education, income, BMI, WC, smoking status, urinary creatinine
			Heart attack: OR = 1.40 (1.11–1.78),	
			Angina: OR = 1.28 (1.09–1.50)	
			CHD: OR = 1.63 (1.18–2.26)	
			CVD: OR = 1.39 (1.18–1.63)	
			Stroke: OR = 0.97 (0.74–1.27)	
Melzer et al. 2010 [40]	MI, angina, CHD, CVD (any diagnoses of MI, angina or CHD); all self-reported doctor diagnoses	BPA continuous	MI, per SD increase:	Age, gender, ethnicity, education, income, BMI, WC, smoking status, urinary creatinine
			2003/04: OR = 1.40 (1.07–1.84)	
			2005/06: OR = 1.39 (1.00–1.94)	
			Pooled 2003/06: OR = 1.32 (1.15–1.52)	
			Angina, per SD increase:	
			2003/04: OR = 1.27 (1.06–1.54)	
			2005/06: OR = 1.16 (0.88–1.53)	
			Pooled 2003/06: OR = 1.24 (1.07-1.43)	
			CHD, per SD increase:	
			2003/04: OR = 1.60 (1.11–2.32)	
			2005/06: OR = 1.33 (1.01–1.75)	
			Pooled 2003/06: OR = 1.42 (1.17–1.72)	
			CVD, per SD increase:	
			2003/04: OR = 1.34 (1.10–1.66)	
			2005/06: OR = 1.18 (0.88–1.59)	
			Pooled 2003/06: OR = 1.26 (1.11–1.44)	
Melzer et al. 2012b [59]	CAD: no, intermediate, severe (assessed by angiography)	BPA continuous	Per SD increase:	Age, sex, BMI category, occupational social class, diabetes status
			Intermediate vs. no CAD: OR = 1.69 (0.98–2.94)	
			Severe vs. no CAD: OR = 1.43 (1.03–1.98)	
Incident CVD (1 publication)				
Melzer et al. 2012a [60]	Incident CAD during follow-up: recorded hospital admission and/or died with CAD as underlying cause	BPA continuous	Per SD increase:	Age, sex, urinary creatinine, education level, occupational group, BMI, cigarette smoking, average of the 2 systolic BP readings, total cholesterol, LDL cholesterol, HDL cholesterol, TG, level of physical activity
			OR = 1.11 (1.00–1.23)	
Prevalent hypertension (3 studies)				
Bae et al. 2012 [54]	Hypertension: systolic BP ≥140 mm Hg or diastolic BP ≥90 mm Hg	Ratio of BPA-to-creatinine in quartiles (μg/g creatinine): Q1: <0.37; Q2: 0.37–0.73; Q3: 0.73–1.33; Q4: >1.33	All participants:	Age, sex, height, weight, date of examination, mean fasting blood glucose, smoking status, current consumption of alcohol
			Q2 vs. Q1: OR = 1.21 (0.80–1.84)	
			Q3 vs. Q1: OR = 1.16 (0.78–1.72)	
			Q4 vs. Q1: OR = 1.27 (0.85–1.88)	
			Stratification by gender: non-significant results in males and females.	
			Significant associations in participants without previous history of hypertension	
			Q2 vs. Q1: OR = 2.23 (1.21–4.12)	
			Q3 vs. Q1: OR = 1.79 (1.01–3.17)	
			Q4 vs. Q1: OR = 2.35 (1.33–4.17)	
Shankar & Teppala 2012 [43]	Hypertension: current blood-pressure-reducing medication use and/or systolic BP >140 mmHg and/or diastolic BP >90 mm Hg	BPA continuous (log-transformed)	OR = 1.11 (1.01–1.22)	Age, sex, race/ethnicity, education categories, smoking, alcohol intake, BMI, diabetes, total cholesterol
		BPA in tertiles (ng/mL): T1: <1.5; T2: 1.5–4.0; T3: >4.0	T2 vs. T1: OR = 1.11 (0.71–1.74)	
			T3 vs. T1: OR = 1.50 (1.12–2.00)	
			p for trend = 0.007	
Shiue et al. 2014 [45]	High BP: systolic BP ≥140 mmHg and diastolic BP ≥90 mmHg	BPA continuous (log-transformed)	Adjusted model: OR = 1.14 (1.00–1.30)	Urinary creatinine, age at examination, sex, ethnicity, BMI
			Weighted model (additionally adjusted for subsample weighting): OR = 1.12 (0.93–1.35)	

*BMI* body mass index; *BP* blood pressure; *BPA* bisphenol A; *CAD* coronary artery disease; *CHD* coronary heart disease; *CVD* cardiovascular disease; *HDL* high density lipoprotein; *LDL* low density lipoprotein; *MI* myocardial infraction; *OR* odds ratio; *SD*: standard deviation; *TG* triglycerides; *WC* waist circumference

All studies except 8 [32, 45, 50, 53, 54, 58, 61, 62] adjusted their statistical models for socioeconomic variables (e.g., education, income, occupation). All U.S. studies were adjusted for race/ethnicity.

Ten out of 33 studies (30 %) adjusted their models for dietary intake such as total energy intake [33, 34, 47, 48, 62], sugar-sweetened soda consumption [38, 64], ‘healthy’ food consumption [49, 61, 62] or unbalanced diet (e.g., fast food or sweet consumption) [49, 64]. Casey & Neidell adjusted for potential BPA sources including consumption of bottled water and canned tuna [37].

#### Quality of individual studies

Detailed results of the quality assessment of individual studies according to our scoring system are reported in the Additional file 1: Table S4. In summary, there were 4 (12 %) studies scored as ‘high-quality’, 27 (82 %) scored as ‘medium-quality’ and 2 (6 %) scored as ‘low-quality’. All ‘high-quality’ studies were longitudinal [60, 61, 63, 64]. Results reported in these ‘high-quality’ studies were mixed or significant. Significant and non-significant results were also reported in ‘medium quality’ and ‘low quality’ studies.

### Diabetes and hyperglycemia

#### Diabetes

Eight cross-sectional studies reported on the relation between uBPA and diabetes [32, 37, 39, 40, 42, 46, 51, 56], and seven supported a positive association (Table 2).

In NHANES, a positive association between uBPA concentrations and self-reported diabetes was reported in the NHANES 2003–04 cycle (OR = 1.39, 95 % CI: 1.21–1.60) [39], but results did not reach significance in the NHANES 2005–06 [40] nor 2007–08 [46] populations. Indeed, Casey & Neidell found significant interactions between uBPA and NHANES cycle [37]. Using 3 different statistical models within NHANES cycles from 2003–04 to 2007–08, they reported a positive association in only the 2003–04 cycle.

In a pooled NHANES 2003–08 dataset, Shankar & Teppala used a definition of diabetes based on fasting glucose level, HbA1c level, non-fasting glucose level and self-reported current use of diabetes medication, and found a significant positive linear trend across uBPA quartiles (*p* = 0.002) [42]. Although both statistically significant, the OR for quartile 4 (Q4) compared to quartile 1 (Q1) was about two times higher in normal weight participants (OR = 3.17) than in overweight/obese participants (OR = 1.56).

No significant association was shown in a Korean cross-sectional study [56]. In a Chinese cross-sectional study, the adjusted OR for type 2 diabetes was statistically significant in the second (OR = 1.30, 95 % CI: 1.03–1.64) and fourth (OR = 1.37, 95 % CI: 1.08–1.74) uBPA quartiles, but not in the third uBPA quartile (OR = 1.09, 95 % CI: 0.86–1.39), compared to the first quartile [51]. Among 131 Iranian participants, Ahmadkhaniha et al. reported a significant adjusted OR of 57 for type 2 diabetes when comparing the participants with uBPA above the median to those with uBPA below the median [32].

One prospective study, among U.S. women from the NHS and the NHSII cohorts, has been published [61]. While a positive association between uBPA and incident diabetes was reported among middle-aged women from the NHSII study (OR = 2.08, 95 % CI: 1.17–3.69, for Q4 vs. Q1), no significant results were found in older women from the NHS study (OR = 0.81, 95 % CI: 0.48–1.38, for Q4 vs. Q1).

#### Prediabetes and hyperglycemia

In one cross-sectional study, there was a positive association between uBPA tertiles and prediabetes, with associations stronger in women and obese subjects [41]. Two cross-sectional studies reported the relation between uBPA and elevated fasting blood glucose, one in children [38], one in adults [34]; neither reported a significant association.

#### Meta-analysis

For prevalent diabetes, the inclusion of the study by Ahmadkhaniha et al. [32] led to highly significant heterogeneity (*p* < 10^−5^ and I^2^ = 94 %); consequently we excluded this study from the meta-analysis. Finally, three cross-sectional studies, all examining uBPA levels in quartiles, were included in the meta-analysis (*n* = 9291) [37, 51, 56]. Pooled ORs (95 % CI) were 1.33 (1.10–1.61), 1.18 (0.97–1.44) and 1.47 (1.21–1.80) in the second, third, and fourth uBPA quartiles compared to the first uBPA quartile. No significant heterogeneity was observed (*p* = 0.88, 0.41 and 0.55 respectively, I^2^ = 0 %). We were unable to conduct a meta-analysis for the other outcomes due to insufficient numbers of studies.

### Anthropometry and adiposity

The relationships between uBPA and measures of anthropometry (e.g., weight, overweight, obesity) or adiposity (e.g., fat mass) were investigated in 16 studies, including 8 studies conducted in children (Table 3).

#### Overweight and obesity

A positive cross-sectional association between uBPA and overweight was reported in 2 studies [49, 64], whereas 5 publications reported no significant associations with overweight [36, 38, 47, 52, 55]. In the only longitudinal study, uBPA concentrations at age 5 were not associated with overweight at age 9 [64]. All but one study found a significant positive cross-sectional association between higher uBPA concentrations and obesity [35, 38, 44, 47, 52, 55], with ORs ranging from 1.50 to 2.57 for Q4 vs. Q1.

#### Waist circumference

The four cross-sectional studies examining the relationship between uBPA and prevalent abdominal obesity in adults showed significant positive results [36, 44, 52, 57]. Eng et al. reported that in children, higher uBPA concentrations were significantly associated with elevated WC-to-height ratio (WHR), but not with elevated WC [38].

Three studies considered WC or WHR as a continuous variable. One showed a positive linear association between daily BPA excretion and WC [58] and another, in women, reported a significant positive correlation between uBPA concentrations and WC [53]. The only study in children found a statistically significant positive association between uBPA and WHR [48].

#### Weight and BMI

Three cross-sectional studies examined the association between uBPA and weight [58] or BMI [50, 53] as continuous variables. Both Chinese studies by Wang et al. [50] and Zhao et al. [53] reported significant positive associations between uBPA and BMI. Galloway et al. [58] showed that daily BPA excretion was significantly and positively associated with BMI and weight in Italian adults.

Among the prospective studies, one reported that early childhood uBPA concentrations were associated with a modest and non-significant reduction in child BMI between 2 and 5 years of age [63], whereas the other found that higher uBPA concentrations were significantly associated with modestly faster weight gain in women [62].

#### Adiposity

In Chinese healthy premenopausal women, fat mass was significantly correlated with uBPA when adjusting for age [53]. In pooled NHANES 2003–10, abnormal body fat in children was not associated with uBPA concentrations in quartiles [38].

#### Meta-analysis

We included 5 cross-sectional studies for overweight [36, 38, 49, 52, 64], including 2 in adults, 3 cross-sectional studies for obesity [38, 44, 52], including 2 in adults, and 4 cross-sectional studies for elevated WC [38, 44, 52, 57], including 3 in adults.

Higher BPA exposure was significantly associated with obesity and elevated WC, and no significant heterogeneity between studies was found (Table 5). There was evidence for overweight only in adults.

**Table 5:** Pooled OR estimates for diabetes, overweight, obesity, elevated waist circumference and hypertension comparing extreme categories of urinary BPA levels (the highest vs. the lowest): random effect models

Outcome	Number of studies	Pooled OR^a^ (95 % CI)	Heterogeneity	
			*p*-value	I^2^ (%)
Prevalent diabetes	3	1.47 (1.21–1.80)	0.55	0
Prevalent overweight				
Total	5	1.21 (0.98–1.50)	0.09	45
Children only	3	1.24 (0.88–1.75)	0.03	62
Adults only	2	1.25 (1.01–1.56)	0.84	0
Prevalent obesity				
Total	3	1.67 (1.41–1.98)	0.44	0
Adults only	2	1.60 (1.32–1.93)	0.54	0
Prevalent elevated WC				
Total	4	1.48 (1.25–1.76)	0.28	21
Adults only	3	1.52 (1.21–1.90)	0.15	47
Prevalent hypertension	2	1.41 (1.12–1.79)	0.50	0

*BPA* bisphenol A; *CI*, confidence interval; *OR* odds ratio; *WC* waist circumference

^a^Using ORs comparing extreme categories of urinary BPA levels (the highest vs. the lowest) summarized in Tables 2, 3 and 4

### Cardiovascular disease and hypertension

#### Cardiovascular disease

Four of the five cross-sectional studies reported a positive linear association between uBPA and CVD (Table 4). An increased risk of self-reported CVD (myocardial infarction, angina, or coronary heart disease [CHD]: alone or combined) was associated with increased concentration of uBPA (OR = 1.39, 95 % CI: 1.18–1.63), but no increased risk of stroke (OR = 0.97, 95 % CI: 0.74–1.27) in NHANES 2003–04 [39]. Melzer et al. then reported similar associations when including NHANES 2005–06 data [40]. LaKind et al. examined NHANES data from 2003–04 to 2009–10 in adults aged ≥20 years, and found no significant associations between self-reported CHD, heart attack and uBPA as a continuous variable in either separate or pooled populations [33]. Casey & Neidell reported significant positive associations between uBPA and CHD in NHANES 2003–04 but results were not consistent in the subsequent 2005/06 and 2007/08 cycles [37]. Melzer et al. studied coronary artery disease (CAD) severity assessed by angiography [59]. Compared to participants without CAD (*n* = 120), uBPA concentrations – per standard deviation (SD) increase – were significantly associated with severe CAD (OR = 1.43, 95 % CI: 1.03–1.98; *n* = 385), and near significantly associated with intermediate CAD (OR = 1.69, 95 % CI: 0.98–2.94; *n* = 86).

The only prospective study, a nested case–control study within the EPIC-Norfolk cohort, reported a positive association between uBPA concentrations and incidence of CAD up to 10 years after BPA measurement, with a significant increase in risk of CAD per SD increase in uBPA: OR = 1.11, 95 % CI = 1.00-1.23 [60].

#### Hypertension

Three cross-sectional studies examined the association between uBPA and hypertension (Table 4). Both Shankar & Teppala (using NHANES 2003–04 data) [43] and Shiue et al. (NHANES 2009–10) [45] showed a positive association between uBPA and hypertension. Bae et al. reported a positive but non-significant association between uBPA and hypertension (OR = 1.27, 95 % CI: 0.85–1.88 for Q4 vs. Q1) in an elderly Korean population, and the association reached significance when the analyses were restricted to participants without a previous history of hypertension (OR = 2.35, 95 % CI: 1.33–4.17) [54].

#### Meta-analysis

Meta-analysis for the CVD and uBPA data was not possible owing to different study designs and use of overlapping NHANES data. For hypertension, we included two cross-sectional studies and the pooled OR was statistically significant (Table 5).

## Discussion

### Summary of evidence

Our analysis presents summarized evidence of BPA exposure and its associations with chronic cardiometabolic disorders including glucose abnormalities, measures of overweight/obesity, CVD and hypertension in humans.

Of the 33 epidemiological studies included, results were generally consistent across cross-sectional studies, with positive associations between uBPA concentrations and diabetes, general obesity, abdominal obesity, CVD and hypertension suggested in 7/8, 6/7, 5/5, 4/5 and 2/3 publications respectively. We were able to conduct outcome-specific meta-analysis including 12 independent studies. While a risk of bias cannot be excluded, the results indicate positive cross-sectional associations between uBPA concentrations and diabetes, general obesity, abdominal obesity and hypertension. Results were significant for overweight in adults but not in children. We were unable to examine uBPA and CVD in meta-analysis due to overlapping data and/or heterogeneity among studies (in terms of study design and definition of outcome).

Among the 5 prospective studies included in this review, 3 showed that uBPA levels were positively associated with incident type 2 diabetes and CAD, and weight gain. While these first prospective results seem to corroborate findings from cross-sectional studies, more prospective data are needed to make the evidence more robust.

### Strengths and limitations of the current review

In recent years, there has been a rapid increase in publications examining the possible relationship between BPA exposure and cardiometabolic health. A systematic review of the epidemiological evidence linking BPA with indicators of obesity, glucose metabolism and CVD, was published by LaKind et al. in 2014 [33]. Further to their results, we have been able to include 11 further publications into this review. We have also calculated pooled ORs for diabetes, overweight, general/abdominal obesity, and hypertension. In their systematic review, LaKind et al. concluded that meta-analysis was not feasible due to the heterogeneity across studies. Nonetheless we were able to identify small but significant groups of cross-sectional studies similar enough to be included in a meta-analysis. However it should be noted that studies included in the meta-analyses can differ with regard to some characteristics, such as outcome ascertainment (especially for diabetes), adjustment variables, and exposure contrast.

As with many reviews of observational data, the risk of publication bias cannot be completely ruled out and it can be hypothesized that studies with significant and positive results are more widely disseminated than those with non-significant and/or negative results. We considered producing funnel plots to explore the presence of publication bias across studies included in the meta-analyses. However, as a rule of thumb, tests for funnel plot asymmetry should not be used when there are fewer than 10 studies in the meta-analysis because test power is usually too low to distinguish chance from real asymmetry [65]. We restricted this review to studies published in English, due to the generalization of the English language in scientific publications. To ascertain if we may have omitted studies not published in English, we ran the same search in PubMed, but limiting the results to non-English languages. We found 12 new articles (5 in Chinese, 3 in Japanese, 2 in Italian, 1 in Spanish, and 1 in Czech), but based on abstract, none of them met the eligibility criteria other than language.

### Strengths and limitations of studies included in the review

Most studies were not designed with environmental exposures in mind. Even though there is evidence for a cross-sectional relation between uBPA and cardiometabolic disorders, results were different across studies, even in different cycles from the same study (NHANES). Depending on the studies, possible explanations could be incomplete adjustment for confounders, measurement error, or differences in study design.

When studying the relation between BPA exposure and cardiometabolic disorders, the statistical models should be properly adjusted for confounding variables. Dietary intake, especially high levels of processed food, has to be considered as a major potential confounding factor for the relationship between uBPA and metabolic disease. It has been suggested that people with diabetes or obesity may consume more food, either in absolute volume or from BPA-containing packaging (plastic containers, polycarbonate drink bottles, tin cans coated with epoxy resin, etc.), which may result in higher uBPA concentrations than non-diabetic or healthy weight individuals. Therefore we can suggest that positive findings observed in cross-sectional studies may be due to dietary sources of BPA exposure that are also important predictors of adiposity. Less than one-third of studies included in this review controlled for diet, mostly *via* total daily energy intake, and only 1 for possible dietary sources of BPA exposure (canned tuna fish and bottled water consumption). Thus, there is a strong need for observational studies controlling for dietary characteristics including potential sources of BPA exposure. Moreover, food and drink in BPA-containing packaging are not necessarily similar in terms of nutritional value (e.g., canned vegetables compared to processed meat, or bottled water compared to sugar-sweetened beverages). Consequently, there is a requirement for data on both dietary patterns (e.g., overall energy intake) along with sources of dietary exposure to BPA, as this helps disentangle real associations from confounding.

Additionally, given the potential correlation of environmental toxicants with each other, exposure to other EDCs may be another confounding factor. Two studies controlled for exposure to other environmental phenols [47] or to phthalates [37]. In both studies, when added to final multivariable models, the authors reported that the other contaminants did not substantially change the associations of uBPA concentrations with the studied outcomes.

Other methodological limitations of studies relate to BPA exposure assessment. BPA concentrations have been mostly determined in a single urine measurement. There is accumulating evidence that a single measurement may not reflect chronic exposure to BPA: regardless of the timing of urine sampling (spot, first morning void, 24 h collection), BPA concentrations were shown, in individual adults, to considerably vary throughout the day and the week [66]. Thus, using only a single uBPA measurement may lead to non-differential exposure misclassification of the participants and bias the estimates. The study by Snijder et al., in pregnant women, showed that using only one uBPA measurement resulted in underestimation of the association between maternal uBPA and fetal growth, which was stronger and significant when several measurements were taken into account [67]. Consequently, it would be better to increase the number of measurements per participant. However, in the context of large-scale epidemiological studies, the substantial cost of the BPA assay is clearly a limitation. Interestingly, most studies report only a single urinary measurement yet the majority show positive associations between uBPA levels and the outcomes of interest.

Another controversy that exists in the BPA literature relates to the lack of knowledge on ‘deep’ compartments, which may serve as potential sources of BPA exposure. Before being metabolized by the liver, BPA is a lipophilic compound. Although it was thought to be rapidly metabolized and excreted, recent data showed a half-life longer than expected, suggesting a potential accumulation in body tissues such as adipose tissue [68]. In agreement with this hypothesis, Fernandez et al. showed, among 20 Spanish women, that unconjugated BPA was detected in adipose tissue in 55 % of the samples [69].

The other key issue in the human data is the lack of prospective data, reflecting the inherent difficulties in performing longitudinal studies, which are costly and time-consuming. Five studies used a prospective design and the main outcomes differ. Although literature in this area is rapidly increasing, the majority of studies published have, and continue to be, of a cross-sectional nature. Indeed, among the 11 studies published between 1 June 2013 and 1 August 2014, only 3 have a prospective design. Nearly half of the publications included in this review relied on NHANES cross-sectional data, including a substantial number of publications using the same study population. Consequently, it is imperative that more prospective epidemiological data is presented, especially with outcomes related to glucose tolerance and anthropometry, which appear to show the most consistent associations. Certainly, given chronic diseases such as diabetes develop over a timespan of many years, timing of BPA measurement would need to occur within an etiologically relevant window before diagnosis of the disease.

Differences in analyses and results contribute to confusion about the association between BPA exposure and cardiometabolic disorders. As Casey & Neidell highlighted, using continuous uBPA, continuous log-transformed uBPA or uBPA in quartiles as exposure variable can lead to lack of consistency in results in a same data set [37]. It therefore seems difficult to draw definitive conclusions from the available data, and publications may be vulnerable to biases given that the same data set can produce significant or non-significant associations according to the different statistical models utilized.

At present, the “gold standard” method for testing BPA is solid-phase extraction coupled with HPLC-MS/MS with peak focusing [13]. Overall, chromatography-based methods are expensive and, thus, not particularly suitable for the large numbers of samples which are required to ensure that analyses are adequately powered to detect any association. Less expensive methods have been proposed, focusing on BPA metabolites (e.g., BPA-glucuronide) [70, 71]. It has been suggested that part of the BPA measured in human samples can be due to contamination during sample collection or laboratory measurements [72, 73], and one of the great advantages of studying BPA metabolites is to avoid these contamination issues. The lower cost of these methods would also allow measuring BPA in larger populations. However, at present, the use of urinary levels of BPA metabolites as surrogates for BPA exposure remains to be validated.

### Future research and implications

Experimental studies have suggested that BPA exposure is associated with abnormal glucose metabolism and insulin resistance. However, data from observational studies of humans supporting an association between BPA and obesity, diabetes and CVD are still too limited in cross-sectional studies to make definitive statements of harm. It is imperative that more prospective studies, with careful measurements of dietary intake, socioeconomic status and urine dilution, are conducted to understand the potential impact BPA exposure in humans will have on the development of chronic disease. Concerning the health outcomes of interest, if associations with overweight/obesity defined according to BMI have been much studied, there are a limited number of studies using more accurate measures of adiposity (such as percent body fat or fat mass). In some studies, analyses were stratified by gender, age, BMI or pre-existing disease. Future studies should continue to explore the existence of populations potentially at greatest risk for BPA-related health effects, in order to confirm or refute these first results. Irrespective of outcome, if confirmed, reasons for differences in these associations will need further exploration. Certainly, no epidemiological study has aimed to identify critical windows of exposure and the risk of incident cardiometabolic disorders. For chronic diseases which have a long latent period, different lag times should be tested. This should feature in future longitudinal studies, especially in paediatric cohorts.

Even in the absence of certainty, there are growing community concerns about the possible deleterious impact of BPA on human health. In agreement with the precautionary principle, regulations have been adopted by a number of countries, prohibiting BPA in baby feeding bottles, in food containers for young children, or in all food and drink packaging, with the aim of reducing human exposure to BPA [74]. If the hypothesised associations are confirmed, this will not only provide evidence to further limit or ban the use of BPA in the food industry, but also advance the impetus to research other health outcomes, and to further explore the impact of a broad range of potential environmental toxins on chronic disease. Lastly, given the recent efforts to reduce BPA exposure, finding safe alternatives to BPA is a very important issue. While BPA can be replaced by structurally similar molecules, such as other bisphenols (e.g., bisphenol S, bisphenol F), initial studies suggest similar endocrine disrupting effects [75], and epidemiological studies should now also consider exposure to BPA-replacement chemicals.

## Conclusion

In summary, we have shown that uBPA at levels found in the general population is associated with increased prevalence of diabetes, general and abdominal obesity, and hypertension. Additional prospective data are needed to ascertain the nature of the relationship between BPA exposure and cardiometabolic disorders. Prospective cohort studies, with carefully collected data on dietary sources of BPA exposure and other potential confounders, as well as repeated uBPA measurements, are indicated and will likely prove useful in filling this gap in the literature and clarifying/explicating these complex relationships.

## Additional file

Additional file 1: Figure S1. Full PubMed search strategy used in the systematic review. **Table S1.** Descriptive characteristics of studies included in the systematic review (*n* = 33 studies). **Table S2.** Studies from the systematic review included and excluded from the meta-analysis and reasons for exclusion. **Table S3.** Overview of studies using data from the National Health and Nutrition Examination Survey (NHANES). **Table S4.** Assessment of the quality of individual studies. **Figure S2.** Individual and pooled OR estimates for diabetes, overweight, obesity, elevated waist circumference and hypertension comparing extreme categories of urinary BPA levels (the highest vs. the lowest): random effect models.

## Abbreviations

ADA: American Diabetes Association
ALT: Alanine aminotransferase
BMI: Body mass index
BP: Blood pressure
BPA: Bisphenol A
CAD: Coronary artery disease
CHAMACOS: Center for the health assessment of mothers and children of Salinas
CHD: Coronary heart disease
CI: Confidence interval
CVD: Cardiovascular disease
EDCs: Endocrine-disrupting chemicals
eGFR: Estimated glomerular filtration rate
EPIC: European prospective investigation into cancer
ER: Estrogen-related receptor
GGT: Gamma glutamyltransferase
HOMA: Homeostasis model of assessment
HOME: Health outcomes and measures of the environment
HDL: High density lipoprotein
HPLC-MS/MS: High performance liquid chromatography–tandem mass spectrometry
HR: Hazard ratio
hsCRP: High-sensitivity C-reactive protein
IQR: Interquartile range
LDL: Low density lipoprotein
LOD: Limit of detection
LOQ: Limit of quantification
NHANES: National health and nutrition examination survey
NHS: Nurses’ health study
OR: Odds ratio
PPAR: Peroxisome proliferation agonist receptor
PRISMA: Preferred reporting items for systematic reviews and meta-analyses
Q1: First quartile
Q4: Fourth quartile
TG: Triglycerides
SD: Standard deviation
SE: Standard error
uBPA: Urinary bisphenol A
WC: Waist circumference
WHR: Waist circumference-to-height ratio
