# Supplementary material for: Bisphenol A and the risk of cardiometabolic disorders: a systematic review with meta-analysis of the epidemiological evidence
Source: Environ Health. 2015 May 31;14:46. doi: 10.1186/s12940-015-0036-5 (PMC4472611; doi:10.1186/s12940-015-0036-5)

ADDITIONAL FILE

Bisphenol A and the risk of cardiometabolic disorders: a systematic review with meta-analysis of the epidemiological evidence

Authors:

Fanny Rancière[1,2]*, Jasmine G. Lyons[3]*, Venurs H.Y. Loh[3], Jérémie Botton[1,2,4], Tamara Galloway[5], Tiange Wang[6], Jonathan E. Shaw[3], Dianna J. Magliano[3]

*These authors contributed equally to this work.

Affiliations:

[1]Inserm, U1153, Epidemiology and Biostatistics Sorbonne Paris Cité Research Centre (CRESS), Early origin of the child's health and development (ORCHAD) Team, Villejuif, France

[2]Univ Paris Descartes, UMR1153, Paris, France

[3]Baker IDI Heart and Diabetes Institute, Department of Clinical Diabetes and Epidemiology, Melbourne, Australia

[4]Univ Paris-Sud, Faculty of Pharmacy, Châtenay-Malabry, France

[5]University of Exeter, College of Life and Environmental Sciences, Department of Biosciences, Exeter, United Kingdom

[6]Shanghai Clinical Center for Endocrine and Metabolic Diseases, Rui Jin Hospital, Shanghai Jiao Tong University School of Medicine, Shanghai, China

Figure S1: Full PubMed search strategy used in the systematic review

(“body mass index”[TIAB] OR “body mass index”[MeSH Terms] OR “overweight”[TIAB] OR “overweight”[MeSH Terms] OR “obesity”[TIAB] OR “obesity”[MeSH Terms] OR “waist circumference”[TIAB] OR “waist circumference”[MeSH Terms] OR “body weight”[TIAB] OR “body weight”[MeSH Terms] OR “abdominal obesity”[TIAB] OR “obesity, abdominal”[MeSH Terms] OR “cardiovascular disease”[TIAB] OR “cardiovascular diseases”[MeSH Terms] OR “coronary heart disease”[TIAB] OR “coronary disease”[MeSH Terms] OR “diabetes”[TIAB] OR “diabetes mellitus”[MeSH Terms] OR “hypertension”[TIAB] OR “hypertension”[MeSH Terms] OR “blood pressure”[TIAB] OR “blood pressure”[MeSH Terms] OR “insulin resistance”[TIAB] OR “insulin resistance”[MeSH Terms] OR “glucose intolerance”[TIAB] OR “glucose intolerance”[MeSH Terms]) AND (“bisphenol A”[TIAB] OR “BPA”[TIAB])

Table S1: Studies from the systematic review included and excluded from the meta-analysis and reasons for exclusion

Health outcome	Reference	Age category	Status	Reason if excluded
**Prevalent diabetes**				
	Lang et al. 2008	Adults	Excluded	Not independent from other NHANES studies
	Melzer et al. 2010	Adults	Excluded	Not independent from other NHANES studies
	Ning et al. 2011	Adults	Included	
	Shankar & Teppala 2011	Adults	Excluded	Not independent from other NHANES studies
	Silver et al. 2011	Adults	Excluded	Not independent from other NHANES studies
	Casey & Neidell 2013	Adults	Included	
	Kim & Park 2013	Adults	Included	
	Ahmadkhaniha et al. 2014	Adults	Excluded	"Outlier" study (heterogeneity)
**Incident diabetes**				
	Sun et al. 2014	Adults	Excluded	Only study for this outcome
**Prevalent prediabetes**				
	Sabanayagam et al. 2013	Adults	Excluded	Only study for this outcome
**Prevalent hyperglycemia**				
	Eng et al. 2013	Children	Excluded	Only study in children for this outcome
	Beydoun et al. 2014	Adults	Excluded	Only study in adults for this outcome
**Prevalent overweight**				
	Carwile & Michels 2011	Adults	Included	
	Kim et al. 2011	Adults	Excluded	Not comparable BPA categorisation (continuous and log-transformed)
	Trasande et al. 2012	Children	Excluded	Not independent from other NHANES studies
	Wang et al. 2012a	Adults	Included	
	Eng et al. 2013	Children	Included	
	Harley et al. 2013	Children	Included	
	Li et al. 2013	Children	Included	
**Prevalent obesity**				
	Carwile & Michels 2011	Adults	Excluded	Not independent from other NHANES studies
	Kim et al. 2011	Adults	Excluded	Not comparable BPA categorisation (continuous and log-transformed)
	Shankar et al. 2012	Adults	Included	
	Trasande et al. 2012	Children	Excluded	Not independent from other NHANES studies
	Wang et al. 2012a	Adults	Included	
	Bhandari et al. 2013	Children	Excluded	Not independent from other NHANES studies
	Eng et al. 2013	Children	Included	
**Prevalent elevated WC**				
	Carwile & Michels 2011	Adults	Excluded	Not independent from other NHANES studies
	Shankar et al. 2012	Adults	Included	
	Wang et al. 2012a	Adults	Included	
	Eng et al. 2013	Children	Included	
	Wells et al. 2013	Children	Excluded	Not independent from other NHANES studies
	Ko et al. 2014	Adults	Included	

Health outcome	Reference	Age category	Status	Reason if excluded
**Other endpoints related to anthropometry or adiposity**				
WC	Galloway et al. 2010	Adults	Excluded	Only study for this outcome
BMI	Wang et al. 2012b	Children	Excluded	Only study for this outcome
Various measurements	Zhao et al. 2012	Adults	Excluded	Not comparable estimates (correlation coefficients)
Abnormal body fat	Eng et al. 2013	Children	Excluded	Only study for this outcome
Change in BMI	Braun et al. 2014	Children	Excluded	Only study for this outcome
Weight change rate	Song et al. 2014	Adults	Excluded	Only study for this outcome
**Prevalent CVD**				
	Lang et al. 2008	Adults	Excluded	Not independent from other NHANES studies
	Melzer et al. 2010	Adults	Excluded	Not independent from other NHANES studies
	Melzer et al. 2012b	Adults	Excluded	Not comparable outcome (disease severity)
	LaKind et al. 2012	Adults	Excluded	Largest NHANES dataset but no other independent study with similar outcomes
	Casey & Neidell 2013	Adults	Excluded	Not independent from other NHANES studies
**Incident CVD**				
	Melzer et al. 2012a	Adults	Excluded	Only study for this outcome
**Prevalent hypertension**				
	Bae et al. 2012	Adults	Included	
	Shankar & Teppala 2012	Adults	Included	
	Shiue et al. 2014	Adults	Excluded	Not comparable BPA categorisation (continuous and log-transformed)

BMI: body mass index; BPA: bisphenol A; CVD: cardiovascular disease; NHANES: National Health and Nutrition Examination Survey; WC: waist circumference.

Table S2: Descriptive characteristics of studies included in the systematic review (n=33 studies)

Reference	Study name, country	Sample size	Sample population	Gender: Female (%)	Race/ethnicity (%)	Age (years)	Urinary BPA assessment method & LOD/LOQ if reported	BPA levels (mean, median, etc.)
Ahmadkhaniha et al. 2014	Iran	239	Case-control study, Shariati Hospital outpatient population	56.9	All Iranian	Mean ± SE:<br>56.6 ± 9.7 in cases<br>46.7 ± 8.5 in controls	Morning spot urine sample<br>Solid-phase extraction coupled to GC-MS<br>LOD=0.1 µg/L<br>LOQ=0.2 µg/L	Median: 0.85 µg/L
Bae et al. 2012	The Korean Elderly Environmental Panel Study, South Korea	521	Panel study, Korean elderly participating in medical examinations ≤5 times during the study period	73.5	All Korean	All aged ≥60<br>Mean in men: 71.3<br>Mean in women: 70.3	Morning spot urine samples after fasting for ≥8 hours<br>HPLC-MS/MS<br>LOD=0.012 µg/L	Mean:<br>1.0 µg/g creatinine in men<br>1.3 µg/g creatinine in women
Beydoun et al. 2014	Pooled NHANES 2005-08, USA	1586	Cross-sectional study, civilian non-institutionalized United States adults	49.7	Non-Hispanic whites: 48.4<br>Non-Hispanic blacks: 20.4<br>Mexican Americans: 19.2<br>Other Hispanics: 7.7<br>Others: 4.3	Age ≥18<br>Mean ± SE: 45.4 ± 0.7	Spot urine sample<br>Solid-phase extraction coupled to HPLC-MS/MS with peak focusing<br>Lower LOD=0.4 ng/mL	Median (IQR): 2.0 (1.0-3.7) ng/mL
Bhandari et al. 2013	Pooled NHANES 2003-08, USA	2200	Cross-sectional study, civilian non-institutionalized United States children	48.5	Non-Hispanic whites: 62.4<br>Non-Hispanic blacks: 14.4<br>Mexican Americans: 12.5<br>Others: 10.7	Range: 6-18 years<br>Mean ± SE: 12.3 ± 0.1	Spot urine sample<br>Solid-phase extraction coupled to HPLC-MS/MS with peak focusing	Mean ± SE: 4.8 ± 0.2 ng/mL
Braun et al. 2014	HOME study, USA	285	Population-based, mother-child prospective cohort study in Cincinnati, Ohio	Not available	Maternal race<br>White: 66.7<br>Black: 27.6<br>Other: 5.7	Mean ± SD at the 2 time points:<br>1.08 ± 0.09<br>2.09 ± 0.08	Up to 2 spot urine samples at around 1 and 2 years of age<br>Solid-phase extraction coupled to HPLC-MS/MS with peak focusing<br>LOD=0.4 ng/mL	Median (IQR): 3.6 (1.8-6.9) ng/mL
Carwile & Michels 2011	Pooled NHANES 2003-06, USA	2747	Cross-sectional study, civilian non-institutionalized United States adults	50.4	Non-Hispanic whites: 70.3<br>Non-Hispanic blacks: 11.8<br>Mexican Americans: 8.1<br>Other Hispanics: 3.9<br>Others: 6.0	Range: 18-74	Spot urine sample<br>Solid-phase extraction coupled to HPLC-MS/MS with peak focusing<br>Lower LOD=0.36 ng/mL in2003-04 and 0.4ng/mL in2005-06	GM controlled for creatinine (IQR): 2.05 (1.18-3.33) µg/g creatinine
Casey & Neidell 2013	NHANES 2003-04, USA	1455	Cross-sectional studies, civilian non-institutionalized United States adults	Not available	Not available	Range: 18-74	Spot urine sample<br>Solid-phase extraction coupled to HPLC-MS/MS with peak focusing<br>LOD=0.36 ng/ml in 2003-04 and 0.40 ng/ml in 2005-06 and 2007-08	Mean=4.78, 4.16, 3.76 ng/mL<br>Median=2.8, 2.1, 2.0 ng/mL<br>in NHANES 2003/04, 2005/06 and 2007/08 respectively
	NHANES 2005-06, USA	1498						
	NHANES 2007-08, USA	1705						
Eng et al. 2013	Pooled NHANES 2003-10, USA	3370	Cross-sectional study, civilian non-institutionalized United States children	49.0	Non-Hispanic whites: 60.8<br>Non-Hispanic blacks: 14.5<br>Mexican Americans: 12.7<br>Other Hispanics: 5.1<br>Others: 6.9	Range: 6-18<br>Mean ± SD: 12.1 ± 3.7	Spot urine sample<br>Solid-phase extraction coupled to HPLC-MS/MS with peak focusing<br>LOD=0.4 ng/mL	Median (IQR): 2.6 (1.3–4.9) ng/mL
Galloway et al. 2010	InCHIANTI Study, Italy	715	Cross-sectional analyses using data from a prospective population-based study of Italian adults	53.6	Not available	Range: 20-74	24-hour urine sample<br>Solid-phase extraction coupled to HPLC-MS/MS with peak focusing<br>LOQ=0.50 ng/mL	GM: 3.59 ng/mL
Harley et al. 2013	CHAMACOS cohort, USA	311	Longitudinal birth cohort study, participants from the Center for the Health Assessment of Mothers and Children of Salinas, California	Not available	Maternal race/ethnicity<br>Latinas : 97.8<br>Non-Latinas, whites : 1.2<br>Others : 1.0	Mean ± SD at the 2 time points:<br>5.1 ± 0.2<br>9.4 ± 0.4	Up to 2 spot urine samples at 5 and 9 years old<br>Solid-phase extraction coupled to HPLC-MS/MS with peak focusing<br>LOD=0.4 µg/L	GM (IQR):<br>2.5 (1.3-4.6) µg/L at 5 years<br>1.5 (0.9-2.8) µg/L at 9 years
Kim et al. 2011	2009 Korean National Human Biomonitoring Survey, South Korea	1870	Population-based, cross-sectional survey representing the adult population residing in the Republic of Korea	57.1	Korean: 100	Range: 18-69<br>Mean: 45.5	Spot urine sample (collection at different times throughout the day)<br>Liquid–liquid extraction and GC/MS<br>LOD=0.05 ng/mL<br>LOQ=0.20 ng/mL	GM (95% CI): 1.90 (1.81–1.99) ng/mL

Reference	Study name, country	Sample size	Sample population	Gender: Female (%)	Race/ethnicity (%)	Age (years)	Urinary BPA assessment method & LOD/LOQ if reported	BPA levels (mean, median, etc.)
Kim & Park 2013	2009 Korean National Human Biomonitoring Survey, South Korea	1210	Population-based, cross-sectional survey representing the adult population residing in the Republic of Korea	58.4	Korean: 100	Range: 40-69<br>Mean: 53.4	Spot urine sample (collection at different times throughout the day)<br>Liquid–liquid extraction coupled to GC-MS<br>LOD=0.05 ng/mL<br>LOQ=0.20 ng/mL	GM (95% CI): 2.03 (1.92 –2.14) ng/mL
Ko et al. 2014	Study on the integrated exposure to hazardous materials for safety control, South Korea	1030	Cross-sectional study, Korean adults	54.8	Korean: 100	Mean ± SD: 44.3 ± 14.6	12-hour urine sample<br>HPLC-MS/MS<br>LOD not reported	Median (min, max): 1.4 (0.2, 198.7) µg/mL
Lakind et al. 2012	NHANES 2003-04, USA	1057	Cross-sectional studies, civilian non-institutionalized United States children and adults	Not available	Not available	Age ≥20 for CHD and heart attack<br>Study excluded for diabetes (age ≥1 or 12)	Spot urine sample<br>Solid-phase extraction coupled to HPLC-MS/MS with peak focusing<br>LOD=0.36 ng/mL for the 2003/04 survey and 0.4 ng/mL for the other three surveys	Not reported
	NHANES 2005-06, USA	1082						
	NHANES 2007-08, USA	1302						
	NHANES 2009-10, USA	1370						
	Pooled NHANES 2003-10	4811						
Lang et al. 2008	NHANES 2003-04, USA	1455	Cross-sectional study, civilian non-institutionalized United States adults	51.8	Non-Hispanic whites: 69.2<br>Non-Hispanic blacks: 11.6<br>Mexican Americans: 8.5<br>Other Hispanics: 4.3<br>Others: 6.4	Range: 18-74	Spot urine sample<br>Solid-phase extraction coupled to HPLC-MS/MS with peak focusing<br>LOD=0.36 ng/mL	Weighted means (95% CI):<br>4.53 (3.98-5.08) ng/mL in men<br>4.66 (3.67-5.65) ng/mL in women
Li et al. 2013	Jiading District, Shanghai, China	1326	Population-based cross-sectional study, children in grades 4–12 from one elementary school, one middle school, and one high school	49.4	Chinese: 100	Mean around 13	Single spot urine sample (non-fasting) between 9 am and 4 pm<br>HPLC-FD<br>LOD=0.31 ng/mL	Not reported
Melzer et al. 2010	NHANES 2003-04, USA	1455	Cross-sectional studies, civilian non-institutionalized United States adults	51.8	In NHANES 2003-04 / 2005-06:<br>Non-Hispanic whites: 69.2 / 70.6<br>Non-Hispanic Black: 11.6 / 12.2<br>Mexican Americans: 8.5 / 8.2<br>Other Hispanics: 4.3 / 3.4<br>Others: 6.4 / 5.5	Range: 18-74	Spot urine sample<br>Solid-phase extraction coupled to HPLC-MS/MS with peak focusing<br>LOD=0.36 ng/mL in 2003-04 and 0.4 ng/mL in 2005-06	GM (95% CI): 2.49 (2.20-2.83) ng/mL in 2003-04, and 1.79 (1.64-1.96) ng/mL in 2005-06
	NHANES 2005-06, USA	1493		51		Range: 18-74		
	Pooled NHANES 2003-06	2948		51.4		Range: 18-74		
Melzer et al. 2012a	EPIC-Norfolk cohort, UK	1619	Case-control study nested in a British prospective population study, adults	35.8	Not available	Range at baseline: 40-74<br>Mean ± SD:<br>63.8 ± 7.3 in controls<br>64.1 ± 7.5 in cases	Spot urine sample<br>Solid-phase extraction coupled to HPLC-MS/MS with peak focusing<br>LOD<0.50 ng/mL<br>LOQ=0.50 ng/mL	GM: 1.3ng/mL
Melzer et al. 2012b	MaGiCAD study, UK	591	Patient population drawn from the EPIC-Norfolk and neighbouring geographical areas, referred to the regional angiography centre at Papworth Hospital, adults	32.0	Not available	Range: 30-95	Spot urine sample<br>Solid-phase extraction coupled to HPLC-MS/MS with peak focusing<br>LOD<0.50 ng/mL<br>LOQ=0.50 ng/mL	Mean (SD): 3.14 (5.96) ng/mL<br>Median (IQR): 1.58 (0.78-3.03) ng/mL
Ning et al. 2011	Songnan, Baoshan District, Shanghai, China	3423	Cross-sectional study, Chinese adults	60.0	Chinese: 100	Age ≥40<br>Median: 59.0	Morning spot urine sample<br>LC-MS/MS<br>0.30 ng/mL	Median (IQR): 0.81 (0.47 –1.43) ng/mL
Sabanayagam et al. 2013	Pooled NHANES 2003-08, USA	3516	Cross-sectional study, civilian non-institutionalized United States adults	52.8	Non-Hispanic whites: 71.4<br>Non-Hispanic blacks: 9.4<br>Mexican Americans: 8.8<br>Others: 10.4	Age ≥20<br>Mean ± SE:<br>43.1±0.5 in men<br>44.6±0.4 in women	Spot urine sample<br>Solid-phase extraction coupled to HPLC-MS/MS with peak focusing<br>Lower LOD=0.36 ng/mL in 2003/04 and 0.4 ng/mL in 2005/06 and 2007/08	Mean ± SD (ng/mL):<br>Men: 2.22 ± 2.82<br>Women: 1.93 ± 3.14
Shankar & Teppala. 2011	Pooled NHANES 2003-08, USA	3967	Cross-sectional study, civilian non-institutionalized United States adults	53	Non-Hispanic whites: 71.5<br>Non-Hispanic blacks: 9.7<br>Mexican Americans: 8.5<br>Others: 10.2	Age >20<br>Mean ± SE:<br>44.3 ± 0.5 in men<br>45.6 ± 0.4 in women	Spot urine sample<br>Solid-phase extraction coupled to HPLC-MS/MS with peak focusing<br>Detection levels of 0.1–2 ng/mL	Mean ± SE (ng/mL)<br>Men: 3.97 ± 0.21<br>Women: 3.90 ± 0.26

Reference	Study name, country	Sample size	Sample population	Gender: Female (%)	Race/ethnicity (%)	Age (years)	Urinary BPA assessment method & LOD/LOQ if reported	BPA levels (mean, median, etc.)
Shankar & Teppala 2012	NHANES 2003-04, USA	1380	Cross-sectional study, civilian non-institutionalized United States adults	49.6	Non-Hispanic whites: 72.3<br>Non-Hispanic blacks: 9.8<br>Mexican Americans and others: 17.8	Age>20<br>Mean ± SE: 46.2 ± 0.5	Spot urine sample<br>Solid-phase extraction coupled to HPLC-MS/MS with peak focusing<br>Detection levels of 0.1–2 ng/mL	Not reported
Shankar et al. 2012	Pooled NHANES 2003-08, USA	3967	Cross-sectional study, civilian non-institutionalized United States adults	51.7	In men / women:<br>Non-Hispanic whites: 71.5 / 70.0<br>Non-Hispanic blacks: 9.7 / 11.5<br>Mexican Americans: 8.5 / 8.0<br>Others: 10.2 / 10.4	Age>20<br>44.3 ± 0.5 in men<br>45.6 ± 0.4 in women	Spot urine sample<br>Solid-phase extraction coupled to HPLC-MS/MS with peak focusing<br>Detection levels of 0.1–2 ng/mL	Mean ± SE:<br>3.97 ±0.21 ng/mL in men<br>3.90 ± 0.26 ng/mL in women
Shiue et al. 2014	NHANES 2009-10, USA	2865	Cross-sectional study, civilian non-institutionalized United States adults	Not available	Not available	Age>20	Spot urine sample<br>Solid-phase extraction coupled to HPLC-MS/MS with peak focusing	Normal BP: 4.69 ± 27.69<br>High BP: 3.69 ± 13.21<br>(No unit reported)
Silver et al. 2011	Pooled NHANES 2003-08, USA	4389	Cross-sectional study, civilian non-institutionalized United States adults	51.5	Non-Hispanic whites: 72.0<br>Non-Hispanic blacks: 10.4<br>Mexican Americans: 7.9<br>Other Hispanics: 4.0<br>Others: 5.6	Age>20<br>Mean: 46.5 ± 0.36	Spot urine sample<br>Solid-phase extraction coupled to HPLC-MS/MS with peak focusing<br>Lower LOD=0.36 ng/mL in 2003/04 and 0.4 ng/mL in 2005/06 and 2007/08	GM (95% CI), ng/mL<br>2.4 (2.1-2.7) in 2003/04<br>1.7 (1.6-1.9) in 2005/06<br>2.0 (1.8-2.1) in 2007/08
Song et al. 2014	Pooled NHS and NHSII cohorts, USA	977	Prospective study, controls from the case-control study by Sun et al (2014), female registered nurses	100	White: 97<br>Other: 3	Range at urine collection:<br>53-79 in NHS<br>32-52 in NHSII	First-morning-void urine sample<br>LC-MS/MS<br>LOD=0.05 ng/mL	Not reported
Sun et al. 2014	NHS cohort, USA	787	Prospective nested case-control study, female registered nurses	100	White: 98<br>Other: 2	Mean ± SD at urine collection: 65.6 ± 6.4	First-morning-void urine sample<br>LC-MS/MS<br>LOD=0.05 ng/mL	Median (IQR), µg/L:<br>NHS cases: 1.5 (1.0-2.8)<br>NHS controls: 1.5 (1.0-2.7)<br>NHSII cases: 2.3 (1.4-3.8)<br>NHSII controls: 2.0 (1.3-3.5)
	NHSII cohort, USA	1154			White: 96<br>Other: 4	Mean ± SD at urine collection: 45.6 ± 4.4		
Trasande et al. 2012	Pooled NHANES 2003-08, USA	2838	Cross-sectional study, civilian non-institutionalized United States children	51.3	Non-Hispanic whites: 62.0<br>Non-Hispanic blacks: 14.9<br>Hispanic-Mexican Americans: 12.2<br>Other Hispanics: 4.6<br>Others: 6.3	Range: 6-19<br>Age 6-11: 42%<br>Age 12-19: 58%	Spot urine sample<br>Solid-phase extraction coupled to HPLC-MS/MS with peak focusing<br>Lower LOD=0.36 ng/mL in 2003-04 and 0.4 ng/mL in 2005-06 and 2007-08	Median (IQR): 2.8 (1.5–5.6) ng/mL
Wang et al. 2012a	Songnan, Baoshan District, Shanghai, China	3390	Cross-sectional study, Chinese adults	60.0	Chinese: 100	Age≥40<br>Mean ± SD: 60.8 ± 9.9	Spot morning urine sample<br>LC-MS/MS<br>Lower LOD=0.30 ng/mL	Median (IQR): 0.81 (0.47-1.43) ng/mL
Wang et al. 2012b	Changning District, Shanghai City, China	259	Cross-sectional study, Chinese school children	49.8	Chinese: 100	Range: 8-15	First morning urine sample<br>Solid-phase extraction coupled with UPLC-MS/MS<br>LOD= 0.07 ng/mL	GM (95% CI): 0.45 (0.37-0.55) ng/mL<br>Median (IQR): 0.60 (0.20-1.37) ng/mL
Wells et al. 2013	Pooled NHANES 2003-10, USA	2836	Cross-sectional study, civilian non-institutionalized United States children	49.1	Non-Hispanic whites: 61.5<br>Non-Hispanic blacks: 14.0<br>Others: 24.5	6-18 years<br>Mean (95% CI): 12.4 (12.2-12.6)	Single-spot urine sample<br>Solid-phase extraction coupled to HPLC-MS/MS with peak focusing	GM (95% CI): 2.6 (2.4-2.7) ng/mL
Zhao et al. 2012	Shanghai City, China	246	Cross-sectional study, healthy premenopausal women with regular menstrual cycles	100	Chinese: 100	Age≥20<br>Mean ± SE: 35.2 ± 0.6	Second morning urine sample<br>LC-MS/MS<br>Lowest LOD=0.3 ng/mL	Mean ± SE: 2.27 ± 0.32 ng/mL

BPA: bisphenol A, CI: confidence interval, CHAMACOS: Center for the Health Assessment of Mothers and Children of Salinas, CHD: coronary heart diseases, EPIC: European Prospective Investigation into Cancer and Nutrition, HOME: Health Outcomes and Measures of the Environment, IQR: interquartile range, MaGiCAD: Metabonomics and Genomics in Coronary Artery Disease, NHANES: National Health and Nutritional Examination Survey, NHS: Nurses' Health Study, NHSII: Nurses' Health Study II, SD: standard deviation, SE: standard error.

Table S3: Overview of studies using data from the National Health and Nutrition Examination Survey (NHANES)

	NHANES cycles and sample sizes				Outcome definition
	2003-04	2005-06	2007-08	2009-10	
**Diabetes**					
Lang et al, 2008	n=1455				Self-report of physician DM diagnosis
Melzer et al, 2010	n=1455	n=1493			Self-report of physician DM diagnosis
Shankar & Teppala, 2011			N=3967		FBG >126 mg/dL or NFBG >200 mg/dL or HbA1c >6.5% or self-report of current use of DM medication
Silver et al, 2011	n=1364	n=1363	n=1662		HbA1c ≥6.5% or self-report of current use of DM medication
Casey & Neidell, 2013	n=1455	n=1498	n=1705		Self-report of physician DM diagnosis
**Prediabetes**					
Sabanayagam et al, 2013			N=4792		FBG=100-125 mg/dL or 2-h GTT=140-199 mg/dL or HbA1c=5.7-6.4%
**Hyperglycemia**					
Eng et al, 2013				N=3370	FBG ≥100 mg/dL
Beydoun et al, 2014			N=1586		FBG ≥100 mg/dL
**Overweight**					
Carwile & Michels, 2011		N=2747			25≤ BMI <30 kg/m^2
Trasande et al, 2009			N=2838		BMI ≥85th percentile for age and gender
Eng et al, 2013				N=3370	BMI ≥85th percentile for age and gender
**Obesity**					
Carwile & Michels, 2011		N=2747			BMI ≥30 kg/m^2
Shankar et al, 2012			N=3967		BMI ≥30 kg/m^2
Trasande et al, 2012			N=2838		BMI ≥95th percentile for age and gender
Bhandari et al, 2013			N=2200		BMI ≥95th percentile for age and gender
Eng et al, 2013				N=3370	BMI ≥95th percentile for age and gender
**Elevated WC**					
Carwile & Michels, 2011		N=2747			WC ≥102 cm in men or ≥88 cm in women
Shankar et al, 2012			N=3967		WC ≥102 cm in men or ≥88 cm in women
Eng et al, 2013				N=3370	WC ≥90th percentile for age and gender / Waist-to-height ratio ≥0.5
Wells et al, 2013				N=2836	Waist-to-height ratio
**Cardiovascular disease**					
Lang et al, 2008	n=1455				Self-report of MI, angina, CHD, CVD (any diagnoses of MI, angina or CHD), stroke
Melzer et al, 2010	n=1455	n=1493			Self-report of MI, angina, CHD, CVD (any diagnoses of MI, angina or CHD), stroke
LaKind et al, 2012	n=1057	n=1082	n=1302	n=1370	Self-report of CHD, heart attack
Casey & Neidell, 2013	n=1455	n=1498	n=1705		Self-report of CHD
**Hypertension**					
Shankar & Teppala, 2012	n=1380				Current anti-HT meds or SBP >140 mm Hg or DBP >90 mm Hg
Shiue et al, 2014				n=2865	SBP ≥140 mm Hg and DBP ≥90 mm Hg

CHD: coronary heart disease, CVD: cardiovascular disease, DBP: diastolic blood pressure, DM: diabetes mellitus, FBG : fasting blood glucose, GTT: glucose tolerance test, HT: hypertension, MI: myocardial infarction, NFBG: non-fasting blood glucose, SBP: systolic blood pressure, WC: waist circumference. n' indicates sample size per cycle and 'N' indicates sample size for pooled cycles.

Children population   Adults population

Table S4: Assessment of the quality of individual studies

Reference	Study design	Population-based study	Outcome assessment	Number of urine samples per participant	Adjustment for dietary intake	Adjustment for socioeconomic variables	Control for urine dilution or renal function	Total score	Overall 'quality'
	CS: 0 pt<br>Longitudinal: 2 pt	No: 0 pt<br>Yes: 1 pt	Self-reported: 0 pt<br>Measured: 1 pt	One: 0 pt<br>At least 2: 1 pt	No: 0 pt<br>Yes: 1 pt	No: 0 pt<br>Yes: 1 pt	No: 0 pt<br>Yes: 1 pt		
Ahmadkhaniha et al. 2014	0	0	1	0	0	0	1	2	Low
Bae et al. 2012	0	0	1	1	0	0	1	3	Medium
Beydoun et al. 2014	0	1	1	0	1	1	1	5	Medium
Bhandari et al. 2013	0	1	1	0	0	1	1	4	Medium
Braun et al. 2014	2	1	1	1	0	1	1	7	High
Carwile & Michels 2011	0	1	1	0	0	1	1	4	Medium
Casey & Neidell 2013	0	1	0	0	1	1	1	4	Medium
Eng et al. 2013	0	1	1	0	1	1	1	5	Medium
Galloway et al. 2010	0	1	1	0	0	0	1	3	Medium
Harley et al. 2013	2	0	1	1	1	1	1	7	High
Kim et al. 2011	0	1	1	0	0	1	1	4	Medium
Kim & Park 2013	0	1	0	0	0	1	1	3	Medium
Ko et al. 2014	0	0	1	0	0	1	1	3	Medium
Lakind et al. 2012	0	1	0	0	1	1	1	4	Medium
Lang et al. 2008	0	1	0	0	0	1	1	3	Medium
Li et al. 2013	0	1	1	0	1	1	0	4	Medium
Melzer et al. 2010	0	1	0	0	0	1	1	3	Medium
Melzer et al. 2012a	2	1	1	0	0	1	1	6	High
Melzer et al. 2012b	0	0	1	0	0	1	1	3	Medium

Reference	Study design	Population-based study	Outcome assessment	Number of urine samples per participant	Adjustment for dietary intake	Adjustment for socioeconomic variables	Control for urine dilution or renal function	Total score	Overall 'quality'
	CS: 0 pt<br>Longitudinal: 2 pt	No: 0 pt<br>Yes: 1 pt	Self-reported: 0 pt<br>Measured: 1 pt	One: 0 pt<br>At least 2: 1 pt	No: 0 pt<br>Yes: 1 pt	No: 0 pt<br>Yes: 1 pt	No: 0 pt<br>Yes: 1 pt		
Ning et al. 2011	0	1	1	0	0	1	1	4	Medium
Sabanayagam et al. 2013	0	1	1	0	0	1	0	3	Medium
Shankar & Teppala 2011	0	1	1	0	0	1	1	4	Medium
Shankar & Teppala 2012	0	1	1	0	0	1	0	3	Medium
Shankar et al. 2012	0	1	1	0	0	1	0	3	Medium
Shiue et al. 2014	0	1	1	0	0	0	1	3	Medium
Silver et al. 2011	0	1	1	0	0	1	1	4	Medium
Song et al. 2014	2	1	0	0	1	0	1	5	Medium
Sun et al. 2014	2	1	1	0	1	0	1	6	High
Trasande et al. 2012	0	1	1	0	1	1	1	5	Medium
Wang et al. 2012a	0	1	1	0	0	1	1	4	Medium
Wang et al. 2012b	0	1	1	0	0	0	1	3	Medium
Wells et al. 2013	0	1	1	0	1	1	1	5	Medium
Zhao et al. 2012	0	0	1	0	0	0	0	1	Low

Figure S2: Individual and pooled OR estimates for diabetes, overweight, obesity, elevated waist circumference and hypertension comparing extreme categories of urinary BPA levels (the highest vs. the lowest): random effect models

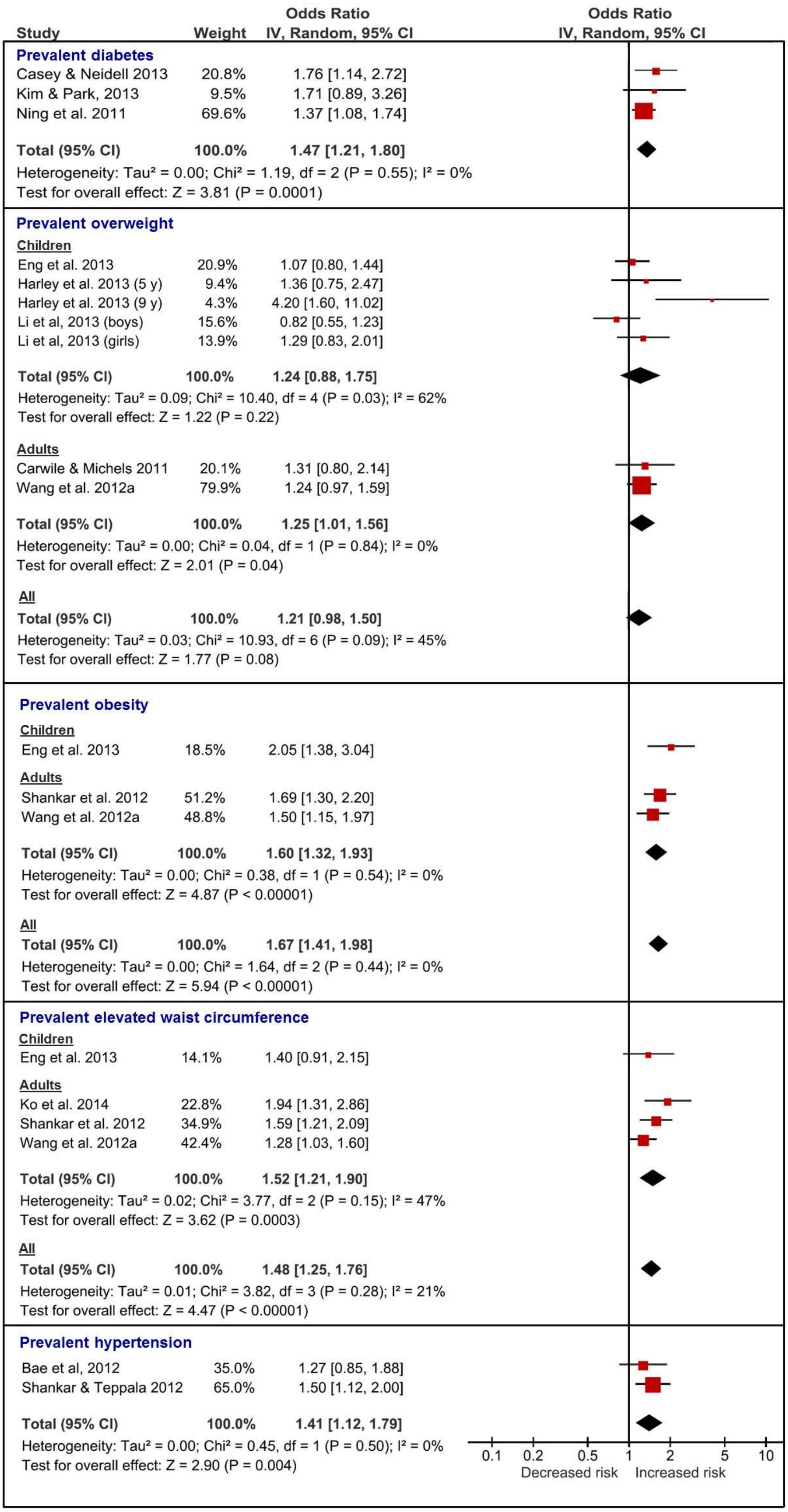

Supplement: Additional file 1: Figure S1. — Full PubMed search strategy used in the systematic review. Table S1. Descriptive characteristics of studies included in the systematic review (n = 33 studies). Table S2. Studies from the systematic review included and excluded from the meta-analysis and reasons for exclusion. Table S3. Overview of studies using data from the National Health and Nutrition Examination Survey (NHANES). Table S4. Assessment of the quality of individual studies. Figure S2. Individual and pooled OR estimates for diabetes, overweight, obesity, elevated waist circumference and hypertension comparing extreme categories of urinary BPA levels (the highest vs. the lowest): random effect models. [file 12940_2015_36_MOESM1_ESM.pdf]
